# Detection of Horse Locomotion Modifications Due to Training with Inertial Measurement Units: A Proof-of-Concept

**DOI:** 10.3390/s22134981

**Published:** 2022-07-01

**Authors:** Benoît Pasquiet, Sophie Biau, Quentin Trébot, Jean-François Debril, François Durand, Laetitia Fradet

**Affiliations:** 1 Plateau technique «Equitation et performance sportive», Institut français du cheval et de l’équitation, Avenue de l’École Nationale d’Équitation, 49411 Saumur, France; sophie.biau@ifce.fr; 2 Equipe Robotique, Biomécanique, Sport, Santé, Institut PPRIME, UPR3346 CNRS Université de Poitiers ENSMA, 86073 Poitiers, France; quentin.trebot@univ-poitiers.fr (Q.T.); laetitia.fradet@univ-poitiers.fr (L.F.); 3 Centre d’Analyse d’Image et Performance Sportive, CREPS de Poitiers, 86580 Vouneuil sous Biard, France; jean-francois.debril@creps-poitiers.sports.gouv.fr (J.-F.D.); francois.durand@creps-poitiers.sports.gouv.fr (F.D.)

**Keywords:** horse locomotion, training effect, inertial measurement units

## Abstract

Detecting fatigue during training sessions would help riders and trainers to optimize their training. It has been shown that fatigue could affect movement patterns. Inertial measurement units (IMUs) are wearable sensors that measure linear accelerations and angular velocities, and can also provide orientation estimates. These sensors offer the possibility of a non-invasive and continuous monitoring of locomotion during training sessions. However, the indicators extracted from IMUs and their ability to show these locomotion changes are not known. The present study aims at defining which kinematic variables and indicators could highlight locomotion changes during a training session expected to be particularly demanding for the horses. Heart rate and lactatemia were measured to attest for the horse’s fatigue following the training session. Indicators derived from acceleration, angular velocities, and orientation estimates obtained from nine IMUs placed on 10 high-level dressage horses were compared before and after a training session using a non-parametric Wilcoxon paired test. These indicators were correlation coefficients (CC) and root mean square deviations (RMSD) comparing gait cycle kinematics measured before and after the training session and also movement smoothness estimates (SPARC, LDLJ). Heart rate and lactatemia measures did not attest to a significant physiological fatigue. However, the statistics show an effect of the training session (*p* < 0.05) on many CC and RMSD computed on the kinematic variables, indicating a change in the locomotion with the training session as well as on SPARCs indicators (*p* < 0.05), and revealing here a change in the movement smoothness both in canter and trot. IMUs seem then to be able to track locomotion pattern modifications due to training. Future research should be conducted to be able to fully attribute the modifications of these indicators to fatigue.

## 1. Introduction

Assessing the athlete, including a sport horse gives meaning to his training. Fatigue and, in particular, muscular fatigue, is a normal outcome of physical exercise. However, delaying the onset of fatigue is a key aim to any training program and is essential for athletic success. [1]. The challenge within a training program is not to cross the limit from which muscular fatigue causes detrimental effects to the musculoskeletal system [2], as fatigue is associated with injury and underperformance [3,4]. Identifying the onset of fatigue and understanding its effects are then essential to optimize training.

Physiological indicators such as heart rate, body temperature, and lactatemia [5] or muscle damage biomarkers [6] provide indications of fatigue during physical exercise. As a consequence of these muscular and physiological effects, movement and locomotion are also modified, which can be seen in the kinematics and spatio-temporal indicators. This has been shown in humans [7,8] and also in horses [9,10]. For horses, their length and stride frequency are thus affected by fatigue. More specifically, difficulty keeping a stable stride frequency [9] and a decrease in stride length [10] have been reported. During endurance events, trot asymmetry was seen to increase as a response to an increase in physical demands [11]. A more recent study also highlighted a decrease in the diagonal step length for thoroughbred horses during canter races. According to the authors, this decrease suggests that horses could not extend their body when fatigued [12].

By combining electromyographic and kinematic analyses, some studies have linked these spatio-temporal modifications to muscular activity changes. Takahashi et al. [13] found that fatigue induced a decrease in the activity of the splenius and brachiocephalicus muscles during canter and trotting exercises, whereas the infraspinatus and deltoid muscles’ activity did not change. This decrease in muscular activity affected the horse’s speed and stride frequency. A change in splenius muscular activity related to fatigue has also been shown to impact head and neck movements [14].

The methods used in these studies were based on optoelectronic systems or electromyography, which are difficult to use in field conditions. Inertial measurement units (IMUs) are wearable sensors that measure 3-dimensional acceleration, angular velocity, and, most of the time, sensor orientation using a combination of accelerometers, gyroscopes, and, most of the time, magnetometers. IMUs have been used effectively in the field to characterize anomalies in horse locomotion since they offer a non-invasive wearable measurement of locomotor indicators. These sensors have been proposed as an aid for lameness detection [15,16,17], for gait classification [18], for horse speed estimation [19], for evaluation of the effect of shoes on break over [20], or for the evaluation of different rehabilitation methods [20]. They also offer an alternative to traditional systems such as optoelectronic systems to identify the phases of locomotion [21,22,23,24,25] or estimate the horse’s protraction and retraction angles [16].

In addition to the question relative to the indicators that could be used to detect fatigue, another question concerns the location of the sensors. A recent study measured the trot movement symmetry of reining horses with three IMUs fixed at the head, wither, and sacrum [15]. Several lameness detection systems are now available on the market (Lameness Locator^®^ (Equinosis, LLC, Columbia, MO, USA), Equigait^®^ (Equigait UK, London, UK)) that are based on three locations, the head, croup, and withers, whereas the Equimoves system^®^ is based on measures located at the four cannons [26]. Thus, no specific sensor location seems to be more favourable to attest for changes in locomotion.

Locomotion indicators modified by fatigue are probably not exhaustive and, as such, it is difficult to determine which of them are the most pertinent for the detection of locomotion changes associated with fatigue. In fact, since IMUs measure acceleration and angular velocities they offer a wide spectrum of kinematic variables. In human studies, they have also been used to assess movement smoothness, which enable the appreciation of defects in motor control [27]. A smoother movement is indeed associated with a skilled behaviour, which represents less effort [28]. Indicators have been developed for the analysis of the smoothness of periodic movements [29]. Among these indicators, the SPARC (for spectral arc length) and the LDLJ (log dimensionless jerk) can be computed from the acceleration and angular velocities measured by IMUs [30]. SPARC has been applied, for example, to IMUs monitoring patients with Parkinson’s disease [31].

The aim of this study is to define which kinematic variables and indicators could be the best to highlight locomotion changes during a training session. To meet these objectives, the evolution of indicators characterising gait derived from acceleration, angular velocities, and orientation estimates extracted from IMUs were compared before and after a training session. The findings from this study could be used to help develop an on-board system to detect in horses the locomotion changes that could be associated with fatigue.

## 2. Materials and Methods

### 2.1. Horses

Ten high level dressage horse/rider combinations competing at the advanced level were involved in this study (1 male, 5 geldings, 4 females; average age of 9.2 ± 2.6 years). These horses were free of clinical signs of lameness.

### 2.2. Material

**Figure 1:**
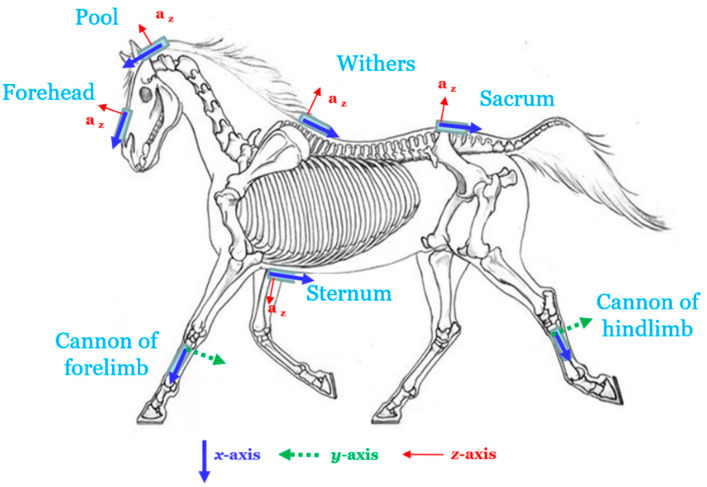
IMUs’ localizations and orientations.

Nine time-synchronised IMUs (Opal, APDM Inc., Portland, OR, USA) were placed on the horse on the forehead and the pool, at the withers, the sternum and the sacrum, and on the distal forelimbs and hindlimbs (Figure 1). They were positioned such that the *x*-axis of the sensor case was aligned with the segment longitudinal axis and the *y*-axis or the *z*-axis was roughly parallel to the segment medio-lateral axis.

The recordings took place in a horse-riding arena. A straight corridor was marked with bars on one side of the arena. The length of this corridor allowed for at least two canter strides to be taken on each hand.

Two monitoring methods were applied to obtain the physiological state of each horse: measurement of the heart rate with a Polar^®^ monitor during the session; measurement of the lactatemia at the end of the work using an Akray^®^ Lactate Pro 2 portable analyser.

### 2.3. Protocol

After a warm-up, the horses were equipped with the IMUs. A first series of locomotion measurements including 2 passages at each gait (trot, canter) was performed in the corridor, one for each direction. Then, the working session was carried out. Immediately at the end of the training session, lactate was taken. Afterwards, a new series of 2 passages at each gait was undertaken in the corridor. Per run, a total of 4 recordings were obtained.

High-level horse training requires individualized planning. Warm-up and working session were decided by the rider, the instruction being that it had to be the most demanding of the sessions scheduled for that period. The volume of the session (gait duration, gait order, and figure repetition) was then entirely managed by the rider. It was not possible to impose the same fatigue protocol to horses of that level with an individualized planning of training.

### 2.4. Data Processing

**Figure 2:**
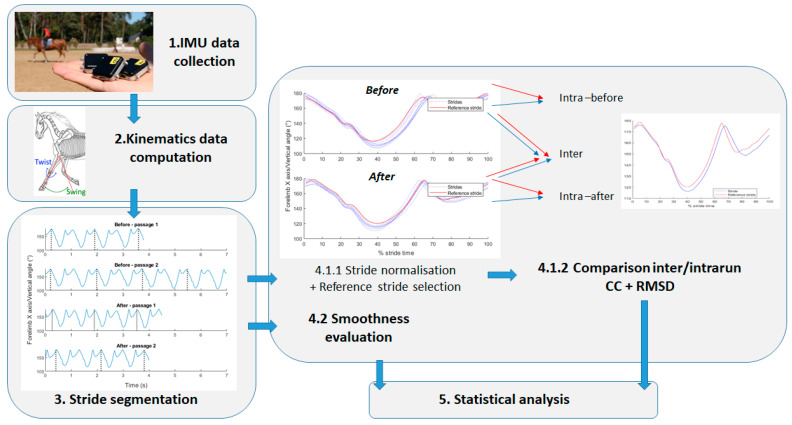
Data processing.

Figure 2 presents data processing.

#### 2.4.1. Kinematics Data

First, the accelerations and angular velocities expressed along the three axes of the IMU reference frame were extracted. With the IMU orientation estimation, we expressed the acceleration in a global coordinate system in order to calculate the components of the acceleration along the vertical axis and in the horizontal plane. In addition, the angle of each axis of the inertial unit was calculated relative to the earth’s vertical. Finally, for the sensors placed on the limbs, a swing–twist decomposition [26,32] was applied. This decomposition provides the rotation angle around the limb axis (twist) and the angle around the lateral axis of the horse (swing).

In total, 13 kinematic signals for the cannons and 11 for the other sensors placed on the head or the sacrum and sternum were recorded.

#### 2.4.2. Stride Segmentation

From the vertical acceleration of the sternum, a stride segmentation of the collected data was taken. For that, we applied a 4th order Butterworth filter to the raw signal. We chose a low-pass filter with a cut-off frequency of 3 Hz, which is a sufficient value to include stride frequency in passband for each gait [33]. A search for consecutive peaks in the filtered signal was performed, imposing a delay greater than 75% of the period between two successive peaks. A manual check on the signal graph was conducted to ensure the correct stride segmentation.

#### 2.4.3. Stride Kinematics’ Comparison

Strides were recorded before and after the training session. A Pchip interpolation provided the same signal length for each stride (100 points). For each one of the kinematics previously described and each gait, strides of a same run (before work or after work) were then grouped. For each group, the stride that minimized the root mean square deviation (RMSD) from this average signal was chosen as the stride of reference.

We then compared each stride to the reference stride of the same run (intra before work or intra after work) or the other run (inter before work vs inter after work), in order to highlight possible modifications in coordination and/or movement amplitude. For this, Pearson correlation coefficient (CC) and root mean square deviation (RMSD) were used.

#### 2.4.4. Smoothness

Two movement smoothness indicators were qualified according to Melendez-Calderon [30]. More specifically, for each stride and each signal, the spectral arc length (SPARC) was calculated, with a cut-off frequency of 10 Hz. Spectrum magnitudes were normalized by their maximum values, to avoid division by zero. The SPARC on angular velocity norm was calculated. In addition, the log dimensionless jerk on acceleration vector (LDLJ-A) after removing gravity was also calculated.

SPARC uses spectrum to quantify sub-movements’ dispersion. It uses magnitude spectrum curve length, until a cut-off frequency. For a signal *v*(*t*), with normalized Fourier magnitude spectrum $\hat{V\left(f\right)}$ and a cut-off frequency $f_{c}$, SPARC is defined as

(1) $$\mathrm{SPARC}=-\int_{0}^{f_{c}}\sqrt{\begin{array}{c} {\left(\frac{1}{f_{c}}\right)}^{2}+{\left(\frac{d\hat{V\left(f\right)}}{df}\right)}^{2\;} \end{array}}\;df$$

LDLJ is an indicator built from the minimum jerk model. For acceleration vector $\vec{a}\left(t\right)$, over time segment $\left[t_{1},t_{2}\right]$, LDLJ-A is computed as

(2) $${\mathrm{LDLJ}}_{a}=-ln\left(\frac{t_{2}-t_{1}}{a_{peak}{}^{2}}\right)\;\int_{t_{1}}^{t_{2}}∥{\frac{d\vec{a}\left(t\right)}{dt}∥}_{2}{}^{2}dt$$

where $a_{peak}$ is acceleration magnitude peak after removing the mean acceleration vector.

All calculations were made using MATLAB 2019b software (The MathWorks Inc., Natick, MA, USA).

### 2.5. Statistical Analysis

To define the effect of the training session, the indicators computed before and after the training session were compared as well as the CC and the RMSD intra versus inter-run. Intra-run values give a null hypothesis as the training session does not cause differences. For these comparisons, we used a non-parametric Wilcoxon paired test. Significance was set to *p* = 0.05. R 4.0.2 software was used to perform statistical analysis.

## 3. Results

### 3.1. Energetic Solicitation during the Training Session

The durations of the training sessions were variable (28 ± 10 min), while the results of HR and lactatemia were homogeneous. The average heart rate of the 10 horses was 112 ± 9 bpm (96 < Fcmav (bpm) < 123) and the blood lactate at the end of the session reached 1 ± 0 mmol/L on average (0.7 < lactate (mmol/L) < 1.1).

### 3.2. Kinematics’ Modifications

The processing of the IMU data made it possible to calculate 834 indicators. The Wilcoxon test provides a *p*-value < 0.01 for 72 indicators and a *p*-value between 0.01 and 0.050 for 168 indicators (see Table S1).

There was no significant difference for the stride durations before training vs after.

Some coefficient correlations computed on variables on the pre-training gait cycles were small. Only the variables for which CC on the pre-training gait cycles were greater than 0.80 were kept. Table 1 presents these significant indicators for CC, Table 2 for SPARC, and Table 3 for RMSD. Appendix A proposes the complete significant results for CC.

Thirteen indicators related to the CC were significantly affected by training for the canter and 12 for the trot. Six indicators related to the SPARC were significantly affected by training for the canter and four for the trot. LDLJ did not provide any significant difference. Finally, 43 indicators related to the RMSD were significantly affected by training for the canter and 58 for the trot.

Eight of them were common to trot and canter for the CC, 39 for RMSD, and none for the SPARC. Among them, both the CC and the RMSD were significantly affected by the training session when computed for: the acceleration along the *x-* and *z*-axes of the forehead sensor, along the x-axis of the pool sensor, as well as for the vertical acceleration of the sternum sensor; the angle between the *x*-axis of the sternum sensor and vertical axis; the angular velocities obtained around the *y*-axis of the sacrum and sternum sensor.

Sixty-six indicators related to the acceleration (obtained in the global or local coordinate system) were modified as a result of the training, whereas 34 were related to angles and 36 to angular velocities.

**Figure 3:**
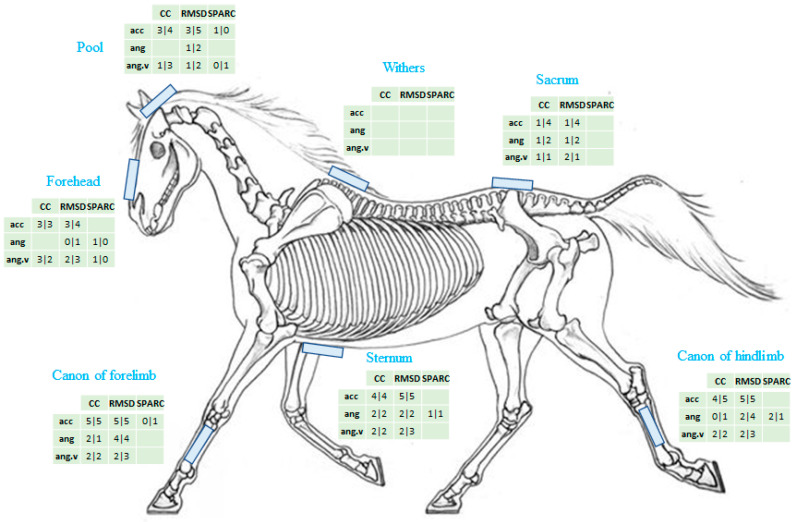
Number of indicators significantly affected by the training session for each sensor location for canter/trot. Here, for the CC, all the significant results are considered.

Regarding the location of the sensor where the significant indicators were seen (Figure 3), when including all the CC significantly affected by the training session, 41 of them were obtained at the sensor located at the forelimb, 38 at the hindlimb cannon, 37 at the sternum, 27 at the pool, 26 at the forehead, and 21 at the sacrum, and none at the withers.

From these results, it emerges, for the trot and the canter, that the CC computed between intra-run strides were superior to the CC computed between inter-run strides.

For the RMSDs, the results go in the same direction, since the RMSD applied to two cycles of a same run was on average lower than the RMSD calculated between two cycles of two different runs.

Regarding the SPARC, at canter, most of the SPARC indicators tend to increase after training, while they tend to decrease at trot.

## 4. Discussion

The aim of the present paper was to define which kinematic indicators extracted from the IMUs’ data could identify locomotion changes following a training session. These indicators could be used in the future to build an on-board system that could help to anticipate detrimental fatigue during training. For this, various locomotion indicators were considered to assess for changes in the kinematics following a training session.

It was expected for the training session to induce fatigue. The riders all interpreted the instruction in the same way and worked with the same objective; however, it was well below the physiological reality. Given the heart rate and blood lactate values, the energy demand (anaerobic and aerobic) was indeed not elevated. The anaerobic threshold expected for the measurements [34] was then not reached, even if the horses were felt to be fatigued according to their riders. In the present study, the maximal blood lactate value measured did not exceed 1.1 mmol/L, which is below the values presented in [35] following two standardised exercise tests with dressage horses and well below the 4 mm/L that can be found following exercises implying running at a specific intensity level [35]. Regarding the heart rate, the maximal heart rate did not exceed 123 bpm in the present study when values over 170 bpm were obtained following the two standardised exercise tests with dressage horses presented in [35].

Despite this, some locomotor indicators were statistically different after vs before the training session. CC and RMSD show a stronger similarity between strides of a same measurement run than between strides of two different runs, which indicates a change in the locomotion following the training session. The change in CC can be interpreted as a modification in the kinematics pattern, whereas a change in the RMSD, in the absence of a change in the pattern, can be seen as a modification in the movement amplitude. We could have expected the forehead and pool kinematics to be more affected than the other kinematics by the training session since modifications in the horse’s head movement has previously been associated with fatigue [14]. In human athletes, it has been shown that the trunk kinematics changed notably and prematurely with fatigue during cycling [36] or running [7]. The absence of a larger effect on the forehead and pool kinematics might be due to the rider, who might influence and reduce the head movements, especially in dressage during which the horse’s posture is evaluated.

The variation in SPARC also reveals a change in the fluidity of movement. The SPARC values were greater in the present study when computed on the angular velocity than in elderly humans during assessments of their gait [31], with values between −2 and −3 obtained in the present study against values around −5 obtained by Beck et al. [31]. The greater smoothness found in the present study might come from many factors such as the fact that horses walk/run on four limbs whereas humans walk/run on two, the “age” of the subjects (adults in the present study vs elderly in [31]), or the potential difference in the IMUs’ noise. At trot, the sternum sensor was impacted regarding this SPARC indicator. It is assumed that this location reflects the body movement because of its rather constant proximity to the centre of gravity at trot, whereas this constant proximity is rather questionable in canter, in particular, because of the pitch of the gait [37]. Thus, the SPARC coefficient calculated from the angular velocity of the sternum seems interesting, and the results show a degradation of the fluidity of the trot after the effort. In canter, SPARC presents an increase in the fluidity, except for the sacrum’s angular velocity. It is known that gluteal muscles and biceps femoris, actors of flexion/extension and abduction/adduction of the hindlimb, can be fatigued at canter [12]. This suggest that fatigue could affect the pelvis movements.

It appears that the indicators could attest to changes in the horse’s locomotion patterns and smoothness following a training session. To the best of our knowledge, no such study has been conducted in horses. In humans, changes in the kinematic range of motion or spatio-temporal indicators have been investigated [38] as well as indicators associated with performance [39].

However, some of the proposed indicators, the CC and RMSD, characterising the evolution in the kinematics might not be the most appropriate to use to develop an on-board device for fatigue detection. This requires the detection on-line of the strides, to save the corresponding stride kinematics, and to compute the CC and RMSD on consecutive kinematic strides and regularly between non-consecutive strides. Approaches based on artificial intelligence could be developed to identify and compare gait cycles during a training session [40].

Because the traditional physiological indicators used to evidence fatigue were moderately affected, a doubt remains: were the observed modifications due to a slight fatigue or were the modifications due to something else such as the technical exercises? If the results are due to the effort itself, then the indicators are very sensitive, which would be promising for the development of a system helping to monitor fatigue.

In fact, the training session was expected to induce a fatigue so important that it would affect these physiological indicators, which was not the case. However, the small impact of the training session on the heart rate and lactate does not necessarily mean that the horses did not experience muscular fatigue. It has been shown that, during sub-maximal exercises, lactatemia does not necessarily significantly increase despite a decrease in the maximal force production [41]. Unfortunately, more elaborated protocols that could attest for a decrease in force production, which is the recognized method to attest for muscular fatigue [42], are not that easy to implement, especially with horses. Nevertheless, other studies measuring, for instance, EMG, as proposed by Takahashi et al. [13] but for locomotion on a treadmill, should be performed to confirm that the modifications seen in the present study can really be attributed to muscular fatigue.

The greatest number of significant variations were obtained in descending order from the fore and hind cannons, then for the sternum, the pool, the forehead, and lastly the sacrum sensors. The sensor located at the withers did not show significant modification, but this is probably due to parasite movements resulting from an inadequate fixation chosen for this sensor. In fact, with the number of indicators being relatively close for the other sensors, it is not possible to really discard a sensor location from the present study.

As a limitation of the present study, the absence of clear fatigue limited the scope of the results. A second limitation is related to the heterogeneous training session chosen by the riders. Another limitation of the study is the lack of information about the warm-up, in terms of duration, intensity, and type (active, passive, specific). It is indeed assumed that these elements improve performance as well as negatively impact it if the content is poorly managed [43]. The warm-up must also be adapted to the needs of the horse according to its lifestyle, its pains, etc. [44]. This is why it is difficult to impose the warm-up on these competitive horses for a protocol of research. However, it would have been relevant to equip the horses with sensors as soon as they had left the stables.

Regarding the kinematics affected by the training session, acceleration and angular velocity-based indicators were numerous even when the kinematics were not expressed in a global reference frame. This can be viewed as positive since these indicators do not require much computation time, which makes it possible to consider the integration of these indicators in an on-board system, to follow the changes in locomotion due to training.

## 5. Conclusions

The aim of the present paper was to define indicators extracted from IMUs’ data that could reveal locomotion changes associated with fatigue in horses. For this, various locomotion indicators were considered to assess for changes in the kinematics following a training session supposed to provoke fatigue in the horses.

If the training session did not seem to induce an important physiological fatigue, some locomotor indicators were statistically different after vs before the training session, indicating that IMUs seem appropriate to track locomotion pattern modifications due to training. Future research should be conducted to be able to fully attribute the modifications of these indicators to fatigue.

## Figures and Tables

**Table 1:** CC significantly affected by the training session. Only the variables for which CC were greater than 0.8 were kept. In grey are highlighted indicators common to canter and trot.

CANTER						TROT					
Variable	Axis	Position	*p*-Value	Before/Intra	Post/Inter	Variable	Axis	Position	*p*-Value	Before/Intra	Post/Inter
Acceleration	*h*	FH	0.031	0.8 (0.06)	0.76 (0.07)	Acceleration	*x*	St	0.008	0.83 (0.06)	0.77 (0.06)
	*x*	FH	0.016	0.94 (0.03)	0.92 (0.04)		*x*	FH	0.047	0.93 (0.03)	0.91 (0.03)
	*x*	Po	0.008	0.92 (0.04)	0.84(0.11)		*x*	Po	0.047	0.93 (0.03)	0.91 (0.03)
	*z*	FH	0.016	0.88 (0.06)	0.81 (0.12)		*z*	Po	0.004	0.94 (0.02)	0.91 (0.03)
	*z*	St	0.039	0.92 (0.04)	0.89 (0.03)		*z*	Sa	0.031	0.96 (0.03)	0.93 (0.05)
	*z*	Po	0.008	0.92 (0.05)	0.87 (0.09)		*v*	St	0.039	0.94 (0.04)	0.93 (0.05)
	*v*	St	0.004	0.91 (0.04)	0.87 (0.04)		*v*	Sa	0.031	0.95 (0.03)	0.93 (0.05)
Angle	*x/v*	Sa	0.031	0.88 (0.25)	0.86 (0.26)	Angle	*x/v*	Sa	0.031	0.82 (0.13)	0.69 (0.24)
	*x/v*	St	0.027	0.96 (0.03)	0.95 (0.04)		*x/v*	FC	0.039	0.84 (0.17)	0.51 (0.5)
Angular Velocity	*y*	FH	0.016	0.87 (0.09)	0.83 (0.11)		*x/v*	St	0.004	0.9 (0.07)	0.82 (0.15)
	*y*	Sa	0.031	0.82 (0.27)	0.76 (0.28)	Angular Velocity	*y*	Sa	0.031	0.84 (0.08)	0.76 (0.12)
	*y*	St	0.02	0.89 (0.07)	0.85 (0.08)		*y*	St	0.008	0.89 (0.07)	0.83 (0.13)
	*y*	Po	0.004	0.82 (0.12)	0.75 (0.2)						

FH: forehead, Po: pool, St: sternum, Sa: sacrum, FC: forelimb cannon, HC: hindlimb cannon. *v*: vertical, *h*: horizontal according to the global reference frame. *x, y, z*: axis in IMU reference frame. *x/v, y/v, z/v*: angle between IMU axis and global reference frame vertical. (cf. Figure 1).

**Table 2:** SPARC significantly affected by the training session. No indicators were common to canter and trot.

CANTER						TROT					
Variable	Axis	Position	*p*-Value	Before/Intra	Post/Inter	Variable	Axis	Position	*p*-Value	Before/Intra	Post/Inter
Acceleration	*v*	Po	0.02	−6.8 (1.6)	−6.2 (1.2)	Acceleration	*v*	FC	0.039	−7.5 (1.8)	−8.5 (1.6)
Angle	Swing	HC	0.004	−2.4 (0)	−2.4 (0)	Angle	Twist	HC	0.004	−2.1 (0.1)	−2.4 (0.1)
	*y/v*	FH	0.031	−2.4 (0)	−2.4 (0)	Angular Velocity	*y*	Po	0.008	−2.7 (0.2)	−2.5 (0.2)
	*z/v*	HC	0.004	−2.4 (0)	−2.4 (0)		*z*	St	0.012	−2.5 (0.2)	−2.7 (0.3)
Angular Velocity	*y*	Sa	0.031	−2.3 (0.2)	−2.4 (0.3)						
	Norm	FH	0.016	−2.3 (0.1)	−2.2 (0.1)						

FH: forehead, Po: pool, St: sternum, Sa: sacrum, FC: forelimb cannon, HC: hindlimb cannon. *v*: vertical, *h*: horizontal according to the global reference frame. *x, y, z*: axis in IMU reference frame. *x/v, y/v, z/v*: angle between IMU axis and global reference frame vertical. (cf. Figure 1).

**Table 3:** RMSD significantly affected by the training session. In grey are highlighted indicators common to canter and trot.

CANTER						TROT					
Variable	Axis	Position	*p*-Value	Before/Intra	Post/Inter	Variable	Axis	Position	*p*-Value	Before/Intra	Post/Inter
Acceleration (m/s^−2^)	*h*	FC	0.047	24.7 (1.8)	29.1 (3.9)	Acceleration (m/s^−2^)	*h*	FC	0.016	12.8 (2.7)	17.4 (4)
	*h*	HC	0.02	22 (2.3)	24.7 (2.5)		*h*	HC	0.016	10.1 (2)	13.6 (4.6)
	*h*	FH	0.031	3.5 (0.9)	4.3 (0.7)		*h*	FH	0.016	2.7 (0.9)	3.2 (1)
	*h*	St	0.004	3.1 (0.4)	3.7 (0.8)		*h*	Sa	0.031	2.8 (0.2)	3.4 (0.3)
	*x*	FC	0.031	24.4 (3)	30.3 (5.1)		*h*	St	0.008	2.7 (0.4)	3.1 (0.5)
	*x*	HC	0.027	21.8 (2.3)	25.5 (4.1)		*h*	Po	0.008	4.5 (1.1)	5.1 (1)
	*x*	FH	0.016	3.2 (0.8)	3.8 (0.9)		*x*	FC	0.02	12.6 (3.3)	17.3 (5.2)
	*x*	Sa	0.031	3.7 (1.1)	4.9 (1)		*x*	HC	0.016	10.9 (2.8)	15.1 (5.3)
	*x*	St	0.008	3.5 (0.5)	4.3 (0.7)		*x*	FH	0.016	3.1 (0.5)	3.6 (0.7)
	*x*	Po	0.004	4.1 (1.2)	5.4 (1.8)		*x*	Sa	0.031	2.2 (0.3)	3 (0.8)
	*y*	FC	0.031	36.2 (4.2)	42.6 (4.6)		*x*	St	0.008	2.7 (0.5)	3.2 (0.6)
	*y*	HC	0.02	28.2 (2.7)	32.9 (4.7)		*x*	Po	0.004	3.3 (0.5)	4.2 (0.8)
	*y*	St	0.012	3.9 (0.5)	4.4 (0.7)		*y*	FC	0.027	21.3 (5.1)	29.6 (9)
	*z*	FC	0.039	15.2 (3.1)	16.7 (2.5)		*y*	HC	0.039	15.2 (4.1)	21.3 (8.1)
	*z*	HC	0.039	13.9 (1.3)	15.4 (1.8)		*y*	FH	0.016	2.2 (0.6)	2.8 (1)
	*z*	FH	0.016	2.3 (1)	2.8 (1)		*y*	St	0.004	2.3 (0.5)	3.1 (1)
	*z*	St	0.039	2.7 (0.5)	3.1 (0.3)		*y*	Po	0.004	2.3 (0.4)	3 (0.8)
	*z*	Po	0.004	2.6 (0.8)	3.5 (1.2)		*z*	FC	0.016	8.7 (1.4)	10.5 (1.6)
	*v*	FC	0.031	29.6 (1.8)	35.8 (5.2)		*z*	HC	0.039	8.3 (1.4)	10.3 (3.2)
	*v*	HC	0.039	28 (2.6)	32.7 (4.6)		*z*	FH	0.016	2 (0.5)	2.7 (0.8)
	*v*	St	0.004	2.8 (0.5)	3.5 (0.5)		*z*	Sa	0.031	2 (0.5)	2.7 (0.6)
	*v*	Po	0.039	6.6 (3)	7.3 (2.7)		*z*	St	0.012	2.1 (0.4)	2.4 (0.5)
Angle (°)	Swing	FC	0.031	8.1 (4.7)	10 (5.8)		*z*	Po	0.004	2.1 (0.4)	2.7 (0.4)
	Twist	FC	0.008	65.6 (7.7)	149.1 (77.9)		*v*	FC	0.031	25.7 (3.1)	29.4 (3.5)
	*x/v*	FC	0.016	15.3 (6)	17.5 (6.6)		*v*	HC	0.039	21.7 (1.4)	25.2 (3.4)
	*x/v*	HC	0.004	10.9 (1.6)	12.8 (2.1)		*v*	Sa	0.031	2.2 (0.4)	2.8 (0.5)
	*x/v*	St	0.008	3.2 (1.2)	4.7 (1.6)		*v*	St	0.02	2.2 (0.3)	2.5 (0.4)
	*y/v*	HC	0.039	16.1 (2.8)	17.9 (2.7)		*v*	Po	0.02	4.4 (2.1)	5 (2)
	*y/v*	Po	0.02	4.7 (1.1)	6 (2)	Angle (°)	Swing	HC	0.02	4.5 (0.9)	7 (2.9)
	*z/v*	FC	0.031	8.1 (4.7)	10 (5.8)		Twist	FC	0.016	88.3 (10.7)	122 (40.7)
	*z/v*	Sa	0.031	2.9 (0.9)	3.3 (0.9)		Twist	HC	0.039	51.3 (18.8)	112.5 (67.3)
	*z/v*	St	0.02	3.4 (1.2)	4.5 (1.6)		*x/v*	FC	0.016	5.6 (2.8)	16.9 (16.4)
Angular Velocity (rad/s)	*x*	FC	0.031	2.5 (0.4)	3.1 (0.6)		*x/v*	HC	0.008	5 (2.8)	9.4 (5.9)
	*x*	HC	0.02	2.2 (0.4)	2.6 (0.6)		*x/v*	St	0.004	2.3 (0.5)	3.7 (1.6)
	*x*	FH	0.047	0.6 (0.1)	0.7 (0.1)		*y/v*	FC	0.016	8.4 (7.7)	24.6 (21.4)
	*x*	St	0.004	0.6(0.1)	0.7(0.1)		*y/v*	Sa	0.031	4.9 (1.3)	6 (1.8)
	*y*	FC	0.02	1.3 (0.2)	1.5 (0.3)		*y/v*	Po	0.02	3.4 (0.8)	4.7 (2.6)
	*y*	HC	0.02	1.2 (0.3)	1.4 (0.4)		*z/v*	FC	0.012	4.9 (2)	6.8 (2.4)
	*y*	FH	0.016	0.5 (0.2)	0.6 (0.2)		*z/v*	HC	0.02	4.5 (0.9)	7 (2.9)
	*y*	Sa	0.031	0.5 (0.3)	0.6 (0.3)		*z/v*	FH	0.031	5.5 (1.2)	8.2 (5)
	*y*	St	0.008	0.4 (0.1)	0.4 (0.1)		*z/v*	Sa	0.031	2.5 (0.7)	3.7 (1.5)
	*y*	Po	0.02	0.5 (0.2)	0.6 (0.2)		*z/v*	St	0.004	2.6 (0.5)	4.4 (1.7)
	*z*	Sa	0.031	0.4 (0.1)	0.5 (0.1)		*z/v*	Po	0.008	4.9 (0.9)	7.7 (3.6)
						Angular Velocity (rad/s)	*x*	FC	0.016	1.7 (0.4)	2.4 (0.9)
							*x*	HC	0.008	1.3 (0.4)	1.9 (0.9)
							*x*	FH	0.016	0.5 (0.1)	0.6 (0.3)
							*x*	St	0.004	0.4 (0.1)	0.6 (0.3)
							*x*	Po	0.004	0.4 (0.1)	0.5 (0.1)
							*y*	FC	0.031	0.8 (0.3)	1.2 (0.4)
							*y*	HC	0.023	0.7 (0.2)	0.9 (0.5)
							*y*	FH	0.031	0.4 (0.1)	0.5 (0.2)
							*y*	Sa	0.031	0.3 (0)	0.3 (0)
							*y*	St	0.008	0.3 (0.1)	0.4 (0.1)
							*z*	FC	0.031	1.6 (1)	3.6 (2.8)
							*z*	HC	0.02	1.4 (0.7)	2.4 (1.7)
							*z*	FH	0.016	0.3 (0.1)	0.4 (0.1)
							*z*	St	0.02	0.3 (0.1)	0.6 (0.4)
							*z*	Po	0.004	0.4 (0.1)	0.5 (0.2)

FH: forehead, Po: pool, St: sternum, Sa: sacrum, FC: forelimb cannon, HC: hindlimb cannon. *v*: vertical, *h*: horizontal according to the global reference frame. *x, y, z*: axis in IMU reference frame. *x/v, y/v, z/v*: angle between IMU axis and global reference frame vertical. (cf. Figure 1).

## Data Availability

Not applicable.

## Supplementary Materials

The following supporting information can be downloaded at: https://www.mdpi.com/article/10.3390/s22134981/s1, Table S1: Complete statistical results.

## Appendix A

Other CC values significantly different between the comparison of cycles from the same run (intra) versus the comparison of cycles measured before and after training (inter) according to the statistical tests. They were not presented in the corpse text of the manuscript because the CC values were smaller than 0.80 for the intra-run.

**Table A1:** CC values significantly different at canter.

CANTER					
Variable	Axis	Position	*p*-Value	Before/Intra	Post/Inter
Acceleration	*h*	FC	0.027	0.28 (0.09)	0.18 (0.14)
	*h*	St	0.004	0.61 (0.14)	0.5 (0.19)
	*x*	FC	0.031	0.31 (0.11)	0.1 (0.1)
	*x*	HC	0.039	0.39 (0.08)	0.26 (0.12)
	*x*	Sa	0.031	0.77 (0.23)	0.67 (0.21)
	*x*	St	0.008	0.72 (0.09)	0.59 (0.07)
	*y*	FC	0.031	0.32 (0.07)	0.16 (0.1)
	*y*	HC	0.039	0.41 (0.07)	0.29 (0.11)
	*z*	FC	0.020	0.24 (0.07)	0.12 (0.07)
	*z*	HC	0.023	0.33 (0.1)	0.23 (0.12)
	*v*	FC	0.016	0.45 (0.12)	0.28 (0.14)
	*v*	HC	0.027	0.36 (0.08)	0.21 (0.1)
	*v*	Po	0.039	0.63 (0.26)	0.56 (0.27)
Angle	Twist	FC	0.008	0.65 (0.09)	0.15 (0.57)
	*x/v*	FC	0.031	0.24 (0.13)	0.13 (0.07)
	*z/v*	St	0.012	0.74 (0.29)	0.66 (0.37)
Angular velocity	*x*	FC	0.027	0.36 (0.08)	0.25 (0.13)
	*x*	HC	0.020	0.38 (0.1)	0.25 (0.1)
	*x*	FH	0.016	0.27 (0.07)	0.1 (0.12)
	*x*	St	0.039	0.2 (0.06)	0.07 (0.13)
	*y*	FC	0.031	0.35 (0.12)	0.23 (0.11)
	*y*	HC	0.016	0.29 (0.11)	0.17 (0.1)
	*z*	FH	0.039	0.28 (0.14)	0.14 (0.24)

FH: forehead, Po: pool, St: sternum, Sa: sacrum, FC: forelimb cannon, HC: hindlimb cannon. *v*: vertical, *h*: horizontal according to the global reference frame. *x, y, z*: axis in IMU reference frame. *x/v, y/v, z/v*: angle between IMU axis and global reference frame vertical. (cf. Figure 1).

**Table A2:** CC values significantly different at trot.

TROT					
Variable	Axis	Position	*p*-Value	Intra	Inter
Acceleration	*h*	FC	0.02	0.62 (0.16)	0.37 (0.25)
	*h*	HC	0.039	0.54 (0.18)	0.22 (0.4)
	*h*	Sa	0.031	0.53 (0.14)	0.33 (0.28)
	*h*	St	0.008	0.6 (0.13)	0.5 (0.15)
	*h*	Po	0.039	0.61 (0.14)	0.51 (0.19)
	*x*	FC	0.016	0.6 (0.2)	0.32 (0.34)
	*x*	HC	0.016	0.62 (0.15)	0.34 (0.33)
	*x*	Sa	0.031	0.76 (0.09)	0.53 (0.28)
	*y*	FC	0.039	0.62 (0.2)	0.32 (0.36)
	*y*	HC	0.004	0.62 (0.18)	0.29 (0.35)
	*y*	FH	0.016	0.55 (0.13)	0.22 (0.37)
	*y*	St	0.008	0.57 (0.21)	0.28 (0.37)
	*y*	Po	0.004	0.63 (0.13)	0.35 (0.39)
	*z*	FC	0.008	0.42 (0.14)	0.18 (0.18)
	*z*	HC	0.004	0.56 (0.13)	0.24 (0.29)
	*z*	FH	0.016	0.75 (0.09)	0.6 (0.21)
	*v*	FC	0.012	0.26 (0.13)	0.04 (0.21)
	*v*	HC	0.02	0.22 (0.13)	0.09 (0.14)
Angle	Twist	HC	0.039	0.66 (0.4)	0.33 (0.67)
	*z/v*	Sa	0.031	0.7 (0.19)	0.55 (0.26)
	*z/v*	St	0.004	0.72 (0.26)	0.66 (0.28)
Angular velocity	*x*	FC	0.004	0.7 (0.12)	0.43 (0.21)
	*x*	HC	0.039	0.74 (0.12)	0.49 (0.38)
	*x*	FH	0.016	0.5 (0.14)	0.11 (0.34)
	*x*	St	0.039	0.67 (0.22)	0.27 (0.57)
	*x*	Po	0.008	0.51 (0.17)	0.22 (0.31)
	*y*	FC	0.02	0.67 (0.23)	0.26 (0.44)
	*y*	HC	0.039	0.63 (0.17)	0.35 (0.43)
	*y*	FH	0.031	0.64 (0.13)	0.48 (0.29)
	*y*	Po	0.02	0.56 (0.18)	0.4 (0.32)
	*z*	Po	0.008	0.51 (0.16)	0.13 (0.37)

FH: forehead, Po: pool, St: sternum, Sa: sacrum, FC: forelimb cannon, HC: hindlimb cannon. *v*: vertical, *h*: horizontal according to the global reference frame. *x, y, z*: axis in IMU reference frame. *x/v, y/v, z/v*: angle between IMU axis and global reference frame vertical. (cf. Figure 1).
