# Supplementary material for: Detection of Horse Locomotion Modifications Due to Training with Inertial Measurement Units: A Proof-of-Concept
Source: Sensors (Basel). 2022 Jul 1;22(13):4981. doi: 10.3390/s22134981 (PMC9269723; doi:10.3390/s22134981)
Supplement: Supplementary file 1 [file sensors-22-04981-s001.zip › sensors-1763097-supplementary.pdf]

Gait	Indicator	Variable	Axis	Position	P.Value	Before/Intra	Post/Inter
Canter	CC	Acceleration	H	FC left	0.027	0.28(0.092)	0.18(0.14)
Canter	CC	Acceleration	H	FC right	0.16	0.27(0.084)	0.1(0.17)
Canter	CC	Acceleration	H	HC left	0.2	0.25(0.067)	0.15(0.099)
Canter	CC	Acceleration	H	HC right	0.13	0.31(0.072)	0.23(0.099)
Canter	CC	Acceleration	H	FH	0.031	0.8(0.058)	0.76(0.07)
Canter	CC	Acceleration	H	Sa	0.094	0.68(0.091)	0.49(0.17)
Canter	CC	Acceleration	H	St	0.0039	0.61(0.14)	0.5(0.19)
Canter	CC	Acceleration	H	Po	0.074	0.76(0.09)	0.72(0.088)
Canter	CC	Acceleration	X	FC left	0.055	0.34(0.048)	0.24(0.1)
Canter	CC	Acceleration	X	FC right	0.031	0.31(0.11)	0.098(0.098)
Canter	CC	Acceleration	X	HC left	0.039	0.39(0.082)	0.26(0.12)
Canter	CC	Acceleration	X	HC right	0.039	0.4(0.09)	0.26(0.12)
Canter	CC	Acceleration	X	FH	0.016	0.94(0.025)	0.92(0.039)
Canter	CC	Acceleration	X	Sa	0.031	0.77(0.23)	0.67(0.21)
Canter	CC	Acceleration	X	St	0.0078	0.72(0.09)	0.59(0.067)
Canter	CC	Acceleration	X	Po	0.0078	0.92(0.038)	0.84(0.11)
Canter	CC	Acceleration	Y	FC left	0.3	0.37(0.065)	0.3(0.12)
Canter	CC	Acceleration	Y	FC right	0.031	0.32(0.074)	0.16(0.098)
Canter	CC	Acceleration	Y	HC left	0.15	0.41(0.09)	0.33(0.1)
Canter	CC	Acceleration	Y	HC right	0.039	0.41(0.075)	0.29(0.11)
Canter	CC	Acceleration	Y	FH	0.16	0.23(0.039)	0.15(0.12)
Canter	CC	Acceleration	Y	Sa	0.16	0.3(0.26)	0.16(0.32)
Canter	CC	Acceleration	Y	St	0.055	0.26(0.084)	0.15(0.19)
Canter	CC	Acceleration	Y	Po	0.5	0.34(0.18)	0.29(0.19)
Canter	CC	Acceleration	Z	FC left	0.02	0.24(0.074)	0.12(0.069)
Canter	CC	Acceleration	Z	FC right	0.031	0.28(0.044)	0.13(0.083)
Canter	CC	Acceleration	Z	HC left	0.023	0.33(0.096)	0.23(0.12)
Canter	CC	Acceleration	Z	HC right	0.012	0.36(0.083)	0.21(0.14)
Canter	CC	Acceleration	Z	FH	0.016	0.88(0.057)	0.81(0.12)
Canter	CC	Acceleration	Z	Sa	0.063	0.93(0.027)	0.86(0.091)
Canter	CC	Acceleration	Z	St	0.039	0.92(0.042)	0.89(0.03)
Canter	CC	Acceleration	Z	Po	0.0078	0.92(0.054)	0.87(0.091)
Canter	CC	Acceleration	V	FC left	0.0078	0.47(0.12)	0.31(0.13)
Canter	CC	Acceleration	V	FC right	0.016	0.45(0.12)	0.28(0.14)
Canter	CC	Acceleration	V	HC left	0.15	0.47(0.051)	0.37(0.12)
Canter	CC	Acceleration	V	HC right	0.027	0.36(0.081)	0.21(0.1)
Canter	CC	Acceleration	V	FH	0.69	0.11(0.12)	0.038(0.18)
Canter	CC	Acceleration	V	Sa	0.063	0.9(0.042)	0.83(0.074)
Canter	CC	Acceleration	V	St	0.0039	0.91(0.041)	0.87(0.041)
Canter	CC	Acceleration	V	Po	0.039	0.63(0.26)	0.56(0.27)
Canter	CC	Angle	Swing	FC left	0.3	0.45(0.15)	0.42(0.15)
Canter	CC	Angle	Swing	FC right	0.11	0.4(0.16)	0.31(0.18)
Canter	CC	Angle	Swing	HC left	0.55	0.28(0.14)	0.24(0.19)
Canter	CC	Angle	Swing	HC right	0.13	0.36(0.16)	0.32(0.17)
Canter	CC	Angle	Twist	FC left	0.0078	0.65(0.091)	0.15(0.57)
Canter	CC	Angle	Twist	FC right	0.078	0.39(0.18)	0.08(0.23)
Canter	CC	Angle	Twist	HC left	0.11	0.51(0.12)	0.26(0.3)
Canter	CC	Angle	Twist	HC right	0.074	0.42(0.16)	0.15(0.39)
Canter	CC	Angle	X/V	FC left	0.73	0.24(0.14)	0.22(0.12)

Canter	CC	Angle	X/V	FC right	0.031	0.24(0.13)	0.13(0.07)
Canter	CC	Angle	X/V	HC left	0.078	0.46(0.11)	0.4(0.11)
Canter	CC	Angle	X/V	HC right	0.57	0.41(0.14)	0.36(0.083)
Canter	CC	Angle	X/V	FH	0.11	0.58(0.21)	0.31(0.52)
Canter	CC	Angle	X/V	Sa	0.031	0.88(0.25)	0.86(0.26)
Canter	CC	Angle	X/V	St	0.027	0.96(0.029)	0.95(0.038)
Canter	CC	Angle	X/V	Po	0.055	0.72(0.21)	0.51(0.51)
Canter	CC	Angle	Y/V	FC left	0.25	0.35(0.089)	0.29(0.15)
Canter	CC	Angle	Y/V	FC right	0.16	0.32(0.14)	0.18(0.15)
Canter	CC	Angle	Y/V	HC left	0.64	0.53(0.1)	0.54(0.099)
Canter	CC	Angle	Y/V	HC right	0.91	0.51(0.13)	0.49(0.095)
Canter	CC	Angle	Y/V	FH	0.078	0.3(0.15)	0.21(0.22)
Canter	CC	Angle	Y/V	Sa	1	0.28(0.34)	0.25(0.37)
Canter	CC	Angle	Y/V	St	0.91	0.13(0.11)	0.061(0.15)
Canter	CC	Angle	Y/V	Po	0.91	0.32(0.16)	0.28(0.24)
Canter	CC	Angle	Z/V	FC left	0.3	0.45(0.15)	0.42(0.15)
Canter	CC	Angle	Z/V	FC right	0.11	0.4(0.16)	0.31(0.18)
Canter	CC	Angle	Z/V	HC left	0.55	0.28(0.14)	0.24(0.19)
Canter	CC	Angle	Z/V	HC right	0.13	0.36(0.16)	0.32(0.17)
Canter	CC	Angle	Z/V	FH	0.22	0.8(0.14)	0.7(0.33)
Canter	CC	Angle	Z/V	Sa	0.094	0.82(0.19)	0.8(0.22)
Canter	CC	Angle	Z/V	St	0.012	0.74(0.29)	0.66(0.37)
Canter	CC	Angle	Z/V	Po	0.43	0.72(0.2)	0.66(0.28)
Canter	CC	Angular Velocity	X	FC left	0.027	0.36(0.076)	0.25(0.13)
Canter	CC	Angular Velocity	X	FC right	0.031	0.36(0.073)	0.15(0.14)
Canter	CC	Angular Velocity	X	HC left	0.2	0.4(0.097)	0.29(0.15)
Canter	CC	Angular Velocity	X	HC right	0.02	0.38(0.095)	0.25(0.1)
Canter	CC	Angular Velocity	X	FH	0.016	0.27(0.065)	0.1(0.12)
Canter	CC	Angular Velocity	X	Sa	0.063	0.27(0.3)	0.15(0.36)
Canter	CC	Angular Velocity	X	St	0.039	0.2(0.063)	0.075(0.13)
Canter	CC	Angular Velocity	X	Po	0.13	0.23(0.18)	0.18(0.21)
Canter	CC	Angular Velocity	Y	FC left	0.02	0.41(0.078)	0.31(0.099)
Canter	CC	Angular Velocity	Y	FC right	0.031	0.35(0.12)	0.23(0.11)
Canter	CC	Angular Velocity	Y	HC left	0.016	0.29(0.11)	0.17(0.1)
Canter	CC	Angular Velocity	Y	HC right	0.055	0.31(0.083)	0.2(0.11)
Canter	CC	Angular Velocity	Y	FH	0.016	0.87(0.092)	0.83(0.11)
Canter	CC	Angular Velocity	Y	Sa	0.031	0.82(0.27)	0.76(0.28)
Canter	CC	Angular Velocity	Y	St	0.02	0.89(0.065)	0.85(0.084)
Canter	CC	Angular Velocity	Y	Po	0.0039	0.82(0.12)	0.75(0.2)
Canter	CC	Angular Velocity	Z	FC left	0.65	0.34(0.064)	0.29(0.099)
Canter	CC	Angular Velocity	Z	FC right	0.16	0.34(0.12)	0.2(0.13)
Canter	CC	Angular Velocity	Z	HC left	0.55	0.51(0.08)	0.48(0.043)
Canter	CC	Angular Velocity	Z	HC right	0.43	0.48(0.11)	0.42(0.11)
Canter	CC	Angular Velocity	Z	FH	0.58	0.31(0.17)	0.27(0.2)
Canter	CC	Angular Velocity	Z	Sa	0.094	0.17(0.051)	0.12(0.063)
Canter	CC	Angular Velocity	Z	St	0.3	0.15(0.06)	0.063(0.13)
Canter	CC	Angular Velocity	Z	Po	0.039	0.28(0.14)	0.14(0.24)
Canter	Duration				0.43	0.62(0.015)	0.63(0.015)
Canter	LDLJ-A			FC left	0.82	-14.0(0.43)	-14.0(0.27)
Canter	LDLJ-A			FC right	0.94	-13.0(1.4)	-14.0(0.28)

Canter	LDLJ-A			HC left	0.55	-13.0(0.43)	-13.0(0.31)
Canter	LDLJ-A			HC right	0.91	-13.0(0.49)	-13.0(0.27)
Canter	LDLJ-A			FH	0.078	-10.0(0.65)	-10.0(0.49)
Canter	LDLJ-A			Sa	0.84	-11.0(0.32)	-11.0(0.26)
Canter	LDLJ-A			St	0.73	-11.0(0.56)	-11.0(0.3)
Canter	LDLJ-A			Po	1	-11.0(0.59)	-10.0(0.55)
Canter	LDLJ-A			FC left	0.36	-10.0(0.15)	-10.0(0.12)
Canter	LDLJ-A			FC right	0.81	-9.9(0.97)	-10.0(0.15)
Canter	LDLJ-A			HC left	1	-9.8(0.22)	-9.8(0.22)
Canter	LDLJ-A			HC right	0.3	-9.9(0.2)	-9.9(0.24)
Canter	LDLJ-A			FH	0.22	-6.7(0.31)	-6.9(0.46)
Canter	LDLJ-A			Sa	0.063	-8.1(0.22)	-8.1(0.25)
Canter	LDLJ-A			St	1	-7.8(0.25)	-7.8(0.29)
Canter	LDLJ-A			Po	0.074	-7.3(0.28)	-7.2(0.29)
Canter	RMSD	Acceleration	H	FC left	0.02	22.0(2.1)	25.0(2.8)
Canter	RMSD	Acceleration	H	FC right	0.047	25.0(1.8)	29.0(3.9)
Canter	RMSD	Acceleration	H	HC left	0.2	18.0(1.9)	20.0(2.4)
Canter	RMSD	Acceleration	H	HC right	0.02	22.0(2.3)	25.0(2.5)
Canter	RMSD	Acceleration	H	FH	0.031	3.5(0.91)	4.3(0.65)
Canter	RMSD	Acceleration	H	Sa	0.063	3.9(0.7)	5.0(0.88)
Canter	RMSD	Acceleration	H	St	0.0039	3.1(0.41)	3.7(0.79)
Canter	RMSD	Acceleration	H	Po	0.055	5.0(1.2)	5.5(1.1)
Canter	RMSD	Acceleration	X	FC left	0.02	24.0(2.4)	27.0(4.5)
Canter	RMSD	Acceleration	X	FC right	0.031	24.0(3.0)	30.0(5.0)
Canter	RMSD	Acceleration	X	HC left	0.023	22.0(2.4)	25.0(3.8)
Canter	RMSD	Acceleration	X	HC right	0.027	22.0(2.3)	25.0(4.1)
Canter	RMSD	Acceleration	X	FH	0.016	3.2(0.8)	3.8(0.91)
Canter	RMSD	Acceleration	X	Sa	0.031	3.7(1.1)	4.9(0.95)
Canter	RMSD	Acceleration	X	St	0.0078	3.5(0.52)	4.3(0.66)
Canter	RMSD	Acceleration	X	Po	0.0039	4.1(1.2)	5.4(1.8)
Canter	RMSD	Acceleration	Y	FC left	0.098	35.0(3.3)	39.0(5.8)
Canter	RMSD	Acceleration	Y	FC right	0.031	36.0(4.2)	43.0(4.6)
Canter	RMSD	Acceleration	Y	HC left	0.11	29.0(2.4)	32.0(2.9)
Canter	RMSD	Acceleration	Y	HC right	0.02	28.0(2.7)	33.0(4.7)
Canter	RMSD	Acceleration	Y	FH	0.078	3.5(0.7)	3.7(0.7)
Canter	RMSD	Acceleration	Y	Sa	0.063	6.4(1.5)	7.4(2.0)
Canter	RMSD	Acceleration	Y	St	0.012	3.9(0.49)	4.4(0.71)
Canter	RMSD	Acceleration	Y	Po	0.2	4.5(1.1)	4.7(1.3)
Canter	RMSD	Acceleration	Z	FC left	0.039	15.0(3.1)	17.0(2.5)
Canter	RMSD	Acceleration	Z	FC right	0.016	14.0(0.89)	16.0(1.3)
Canter	RMSD	Acceleration	Z	HC left	0.039	14.0(1.3)	15.0(1.8)
Canter	RMSD	Acceleration	Z	HC right	0.012	15.0(2.0)	17.0(3.5)
Canter	RMSD	Acceleration	Z	FH	0.016	2.3(1.0)	2.8(0.97)
Canter	RMSD	Acceleration	Z	Sa	0.063	2.6(0.42)	3.9(1.1)
Canter	RMSD	Acceleration	Z	St	0.039	2.6(0.52)	3.1(0.32)
Canter	RMSD	Acceleration	Z	Po	0.0039	2.6(0.83)	3.5(1.2)
Canter	RMSD	Acceleration	V	FC left	0.0039	31.0(3.0)	36.0(3.1)
Canter	RMSD	Acceleration	V	FC right	0.031	30.0(1.8)	36.0(5.1)
Canter	RMSD	Acceleration	V	HC left	0.055	28.0(1.7)	31.0(4.3)
Canter	RMSD	Acceleration	V	HC right	0.039	28.0(2.6)	33.0(4.6)

Canter	RMSD	Acceleration	V	FH	0.58	11.0(1.9)	12.0(1.8)
Canter	RMSD	Acceleration	V	Sa	0.063	3.1(0.69)	4.4(0.86)
Canter	RMSD	Acceleration	V	St	0.0039	2.8(0.49)	3.5(0.54)
Canter	RMSD	Acceleration	V	Po	0.039	6.6(3.0)	7.3(2.7)
Canter	RMSD	Angle	Swing	FC left	0.0078	7.2(1.4)	8.1(1.9)
Canter	RMSD	Angle	Swing	FC right	0.031	8.1(4.7)	10.0(5.8)
Canter	RMSD	Angle	Swing	HC left	0.31	5.3(1.5)	5.8(1.9)
Canter	RMSD	Angle	Swing	HC right	0.074	4.9(1.7)	5.8(1.5)
Canter	RMSD	Angle	Twist	FC left	0.0078	66.0(7.7)	150.0(78.0)
Canter	RMSD	Angle	Twist	FC right	0.22	79.0(20.0)	96.0(20.0)
Canter	RMSD	Angle	Twist	HC left	0.11	51.0(13.0)	97.0(64.0)
Canter	RMSD	Angle	Twist	HC right	0.098	75.0(15.0)	93.0(18.0)
Canter	RMSD	Angle	X/V	FC left	0.13	13.0(2.0)	14.0(1.6)
Canter	RMSD	Angle	X/V	FC right	0.016	15.0(6.0)	18.0(6.6)
Canter	RMSD	Angle	X/V	HC left	0.055	12.0(1.5)	14.0(2.0)
Canter	RMSD	Angle	X/V	HC right	0.0039	11.0(1.6)	13.0(2.0)
Canter	RMSD	Angle	X/V	FH	0.16	5.3(3.2)	5.8(2.9)
Canter	RMSD	Angle	X/V	Sa	0.094	3.1(0.97)	3.5(1.0)
Canter	RMSD	Angle	X/V	St	0.0078	3.2(1.2)	4.7(1.6)
Canter	RMSD	Angle	X/V	Po	0.16	5.3(2.4)	7.9(5.4)
Canter	RMSD	Angle	Y/V	FC left	0.055	23.0(2.6)	26.0(2.8)
Canter	RMSD	Angle	Y/V	FC right	0.078	23.0(3.2)	28.0(4.4)
Canter	RMSD	Angle	Y/V	HC left	0.46	17.0(2.6)	18.0(1.9)
Canter	RMSD	Angle	Y/V	HC right	0.039	16.0(2.8)	18.0(2.7)
Canter	RMSD	Angle	Y/V	FH	0.078	4.4(0.89)	5.8(1.3)
Canter	RMSD	Angle	Y/V	Sa	0.31	4.8(1.9)	5.2(1.6)
Canter	RMSD	Angle	Y/V	St	0.3	6.1(1.0)	6.5(0.75)
Canter	RMSD	Angle	Y/V	Po	0.02	4.7(1.1)	6.0(2.0)
Canter	RMSD	Angle	Z/V	FC left	0.0078	7.2(1.4)	8.1(1.9)
Canter	RMSD	Angle	Z/V	FC right	0.031	8.1(4.7)	10.0(5.8)
Canter	RMSD	Angle	Z/V	HC left	0.31	5.3(1.5)	5.8(1.9)
Canter	RMSD	Angle	Z/V	HC right	0.074	4.9(1.7)	5.8(1.5)
Canter	RMSD	Angle	Z/V	FH	0.16	5.7(3.3)	8.2(4.2)
Canter	RMSD	Angle	Z/V	Sa	0.031	2.9(0.92)	3.3(0.85)
Canter	RMSD	Angle	Z/V	St	0.02	3.4(1.2)	4.5(1.6)
Canter	RMSD	Angle	Z/V	Po	0.074	5.5(2.6)	7.6(4.5)
Canter	RMSD	Angular Velocity	X	FC left	0.02	2.6(0.64)	2.8(0.63)
Canter	RMSD	Angular Velocity	X	FC right	0.031	2.5(0.41)	3.1(0.6)
Canter	RMSD	Angular Velocity	X	HC left	0.078	2.4(0.36)	2.8(0.59)
Canter	RMSD	Angular Velocity	X	HC right	0.02	2.2(0.4)	2.6(0.63)
Canter	RMSD	Angular Velocity	X	FH	0.047	0.62(0.14)	0.68(0.13)
Canter	RMSD	Angular Velocity	X	Sa	0.063	1.3(0.51)	1.6(0.61)
Canter	RMSD	Angular Velocity	X	St	0.0039	0.57(0.1)	0.65(0.1)
Canter	RMSD	Angular Velocity	X	Po	0.055	0.66(0.22)	0.7(0.27)
Canter	RMSD	Angular Velocity	Y	FC left	0.02	1.3(0.24)	1.5(0.28)
Canter	RMSD	Angular Velocity	Y	FC right	0.016	1.2(0.15)	1.4(0.12)
Canter	RMSD	Angular Velocity	Y	HC left	0.0078	1.2(0.23)	1.3(0.26)
Canter	RMSD	Angular Velocity	Y	HC right	0.02	1.2(0.26)	1.4(0.35)
Canter	RMSD	Angular Velocity	Y	FH	0.016	0.51(0.19)	0.59(0.22)
Canter	RMSD	Angular Velocity	Y	Sa	0.031	0.45(0.29)	0.56(0.26)

Canter	RMSD	Angular Velocity	Y	St	0.0078	0.37(0.076)	0.43(0.089)
Canter	RMSD	Angular Velocity	Y	Po	0.02	0.49(0.16)	0.56(0.2)
Canter	RMSD	Angular Velocity	Z	FC left	0.13	4.7(0.59)	5.2(0.42)
Canter	RMSD	Angular Velocity	Z	FC right	0.078	4.7(0.71)	5.6(0.63)
Canter	RMSD	Angular Velocity	Z	HC left	0.38	3.1(0.39)	3.3(0.39)
Canter	RMSD	Angular Velocity	Z	HC right	0.055	3.1(0.39)	3.5(0.56)
Canter	RMSD	Angular Velocity	Z	FH	1	0.52(0.15)	0.54(0.18)
Canter	RMSD	Angular Velocity	Z	Sa	0.031	0.42(0.079)	0.46(0.09)
Canter	RMSD	Angular Velocity	Z	St	0.074	0.61(0.059)	0.68(0.1)
Canter	RMSD	Angular Velocity	Z	Po	0.2	0.52(0.1)	0.56(0.12)
Canter	SPARC	Acceleration	H	FC left	0.43	-4.6(0.32)	-4.5(0.39)
Canter	SPARC	Acceleration	H	FC right	0.58	-4.7(0.55)	-4.9(0.42)
Canter	SPARC	Acceleration	H	HC left	0.95	-4.1(0.4)	-4.1(0.2)
Canter	SPARC	Acceleration	H	HC right	0.36	-5.4(0.69)	-5.6(0.63)
Canter	SPARC	Acceleration	H	FH	0.94	-4.4(0.44)	-4.4(0.34)
Canter	SPARC	Acceleration	H	Sa	0.69	-4.2(0.13)	-4.1(0.33)
Canter	SPARC	Acceleration	H	St	0.3	-4.2(0.43)	-4.3(0.32)
Canter	SPARC	Acceleration	H	Po	0.82	-4.4(0.51)	-4.5(0.32)
Canter	SPARC	Acceleration	X	FC left	0.16	-6.6(1.1)	-6.9(1.2)
Canter	SPARC	Acceleration	X	FC right	0.69	-6.3(1.1)	-6.9(1.4)
Canter	SPARC	Acceleration	X	HC left	0.15	-6.8(0.59)	-7.0(0.73)
Canter	SPARC	Acceleration	X	HC right	0.25	-6.8(1.1)	-7.0(0.88)
Canter	SPARC	Acceleration	X	FH	0.58	-5.1(0.53)	-5.3(0.8)
Canter	SPARC	Acceleration	X	Sa	0.84	-9.9(1.1)	-10.0(1.1)
Canter	SPARC	Acceleration	X	St	0.5	-9.9(2.3)	-10.0(2.1)
Canter	SPARC	Acceleration	X	Po	0.57	-7.6(1.4)	-7.6(1.1)
Canter	SPARC	Acceleration	Y	FC left	1	-9.6(1.3)	-9.6(1.3)
Canter	SPARC	Acceleration	Y	FC right	0.94	-9.1(1.8)	-9.6(1.4)
Canter	SPARC	Acceleration	Y	HC left	0.55	-8.8(0.65)	-9.1(0.73)
Canter	SPARC	Acceleration	Y	HC right	0.43	-9.0(1.2)	-9.2(0.64)
Canter	SPARC	Acceleration	Y	FH	0.69	-11.0(2.1)	-11.0(1.0)
Canter	SPARC	Acceleration	Y	Sa	0.84	-11.0(1.7)	-10.0(0.66)
Canter	SPARC	Acceleration	Y	St	0.82	-11.0(2.2)	-10.0(2.6)
Canter	SPARC	Acceleration	Y	Po	0.73	-9.3(2.1)	-9.2(1.9)
Canter	SPARC	Acceleration	Z	FC left	0.3	-11.0(3.3)	-10.0(2.1)
Canter	SPARC	Acceleration	Z	FC right	0.69	-11.0(4.3)	-10.0(2.8)
Canter	SPARC	Acceleration	Z	HC left	0.84	-12.0(1.2)	-11.0(1.2)
Canter	SPARC	Acceleration	Z	HC right	0.82	-11.0(2.1)	-11.0(1.2)
Canter	SPARC	Acceleration	Z	FH	0.47	-9.7(2.8)	-9.2(2.0)
Canter	SPARC	Acceleration	Z	Sa	1	-4.8(0.29)	-4.9(0.21)
Canter	SPARC	Acceleration	Z	St	1	-4.6(0.35)	-4.7(0.39)
Canter	SPARC	Acceleration	Z	Po	0.91	-5.3(0.7)	-5.2(0.96)
Canter	SPARC	Acceleration	V	FC left	0.098	-8.2(0.87)	-9.1(1.5)
Canter	SPARC	Acceleration	V	FC right	0.38	-7.5(2.1)	-8.3(1.6)
Canter	SPARC	Acceleration	V	HC left	0.38	-11.0(0.95)	-12.0(1.2)
Canter	SPARC	Acceleration	V	HC right	1	-7.5(0.97)	-7.6(1.2)
Canter	SPARC	Acceleration	V	FH	0.94	-5.6(1.2)	-5.7(1.7)
Canter	SPARC	Acceleration	V	Sa	0.69	-5.1(0.33)	-5.0(0.43)
Canter	SPARC	Acceleration	V	St	0.82	-4.7(0.38)	-4.8(0.42)
Canter	SPARC	Acceleration	V	Po	0.02	-6.8(1.6)	-6.2(1.2)

Canter	SPARC	Angle	Swing	FC left	0.13	-2.4(0.091)	-2.5(0.094)
Canter	SPARC	Angle	Swing	FC right	0.22	-2.4(0.087)	-2.4(0.053)
Canter	SPARC	Angle	Swing	HC left	0.84	-2.4(0.063)	-2.4(0.049)
Canter	SPARC	Angle	Swing	HC right	0.055	-2.4(0.044)	-2.4(0.072)
Canter	SPARC	Angle	Twist	FC left	0.91	-2.7(0.26)	-2.7(0.35)
Canter	SPARC	Angle	Twist	FC right	0.3	-2.8(0.38)	-2.8(0.29)
Canter	SPARC	Angle	Twist	HC left	0.74	-2.5(0.12)	-2.5(0.099)
Canter	SPARC	Angle	Twist	HC right	0.57	-2.6(0.25)	-2.5(0.12)
Canter	SPARC	Angle	X/V	FC left	0.074	-2.6(0.09)	-2.7(0.094)
Canter	SPARC	Angle	X/V	FC right	0.69	-2.6(0.13)	-2.6(0.12)
Canter	SPARC	Angle	X/V	HC left	0.64	-2.5(0.12)	-2.5(0.1)
Canter	SPARC	Angle	X/V	HC right	0.43	-2.5(0.083)	-2.5(0.098)
Canter	SPARC	Angle	X/V	FH	0.078	-2.4(0.032)	-2.5(0.017)
Canter	SPARC	Angle	X/V	Sa	0.094	-2.4(0.026)	-2.5(0.034)
Canter	SPARC	Angle	X/V	St	0.2	-2.4(0.068)	-2.5(0.046)
Canter	SPARC	Angle	X/V	Po	0.5	-2.4(0.089)	-2.4(0.041)
Canter	SPARC	Angle	Y/V	FC left	0.2	-2.7(0.21)	-2.8(0.15)
Canter	SPARC	Angle	Y/V	FC right	1	-2.7(0.19)	-2.8(0.16)
Canter	SPARC	Angle	Y/V	HC left	0.55	-2.6(0.1)	-2.6(0.083)
Canter	SPARC	Angle	Y/V	HC right	0.43	-2.7(0.21)	-2.8(0.13)
Canter	SPARC	Angle	Y/V	FH	0.38	-2.5(0.043)	-2.5(0.037)
Canter	SPARC	Angle	Y/V	Sa	1	-2.4(0.043)	-2.4(0.047)
Canter	SPARC	Angle	Y/V	St	0.36	-2.4(0.041)	-2.4(0.039)
Canter	SPARC	Angle	Y/V	Po	0.65	-2.4(0.062)	-2.4(0.082)
Canter	SPARC	Angle	Z/V	FC left	0.13	-2.4(0.091)	-2.5(0.094)
Canter	SPARC	Angle	Z/V	FC right	0.22	-2.4(0.087)	-2.4(0.053)
Canter	SPARC	Angle	Z/V	HC left	0.84	-2.4(0.063)	-2.4(0.049)
Canter	SPARC	Angle	Z/V	HC right	0.055	-2.4(0.044)	-2.4(0.072)
Canter	SPARC	Angle	Z/V	FH	0.47	-2.5(0.13)	-2.4(0.072)
Canter	SPARC	Angle	Z/V	Sa	0.22	-2.9(0.55)	-2.8(0.57)
Canter	SPARC	Angle	Z/V	St	0.16	-2.4(0.04)	-2.5(0.055)
Canter	SPARC	Angle	Z/V	Po	0.36	-2.6(0.22)	-2.5(0.22)
Canter	SPARC	Angular Velocity	X	FC left	0.3	-12.0(2.1)	-11.0(1.3)
Canter	SPARC	Angular Velocity	X	FC right	0.38	-12.0(2.8)	-12.0(1.5)
Canter	SPARC	Angular Velocity	X	HC left	0.25	-12.0(2.2)	-12.0(1.1)
Canter	SPARC	Angular Velocity	X	HC right	0.36	-12.0(2.3)	-12.0(1.1)
Canter	SPARC	Angular Velocity	X	FH	0.58	-12.0(2.9)	-11.0(2.1)
Canter	SPARC	Angular Velocity	X	Sa	1	-11.0(2.8)	-9.9(2.1)
Canter	SPARC	Angular Velocity	X	St	0.13	-9.0(1.2)	-9.5(1.2)
Canter	SPARC	Angular Velocity	X	Po	0.57	-9.7(1.5)	-9.3(1.7)
Canter	SPARC	Angular Velocity	Y	FC left	0.65	-8.4(1.4)	-8.1(1.0)
Canter	SPARC	Angular Velocity	Y	FC right	0.47	-8.9(2.7)	-8.4(1.8)
Canter	SPARC	Angular Velocity	Y	HC left	0.95	-11.0(1.7)	-11.0(2.4)
Canter	SPARC	Angular Velocity	Y	HC right	0.57	-10.0(1.9)	-11.0(1.6)
Canter	SPARC	Angular Velocity	Y	FH	0.16	-7.0(2.2)	-6.6(1.8)
Canter	SPARC	Angular Velocity	Y	Sa	0.31	-6.8(1.0)	-7.1(1.3)
Canter	SPARC	Angular Velocity	Y	St	0.91	-6.5(0.45)	-6.4(0.74)
Canter	SPARC	Angular Velocity	Y	Po	0.57	-7.0(1.8)	-7.4(2.0)
Canter	SPARC	Angular Velocity	Z	FC left	1	-6.7(0.66)	-6.7(0.72)
Canter	SPARC	Angular Velocity	Z	FC right	0.81	-6.4(0.9)	-6.7(0.56)

Canter	SPARC	Angular Velocity	Z	HC left	0.46	-5.9(0.63)	-6.1(0.47)
Canter	SPARC	Angular Velocity	Z	HC right	0.82	-6.0(0.69)	-6.0(0.35)
Canter	SPARC	Angular Velocity	Z	FH	0.94	-8.3(1.4)	-8.0(0.84)
Canter	SPARC	Angular Velocity	Z	Sa	0.84	-9.9(1.3)	-9.9(1.9)
Canter	SPARC	Angular Velocity	Z	St	0.82	-8.4(1.6)	-8.4(2.1)
Canter	SPARC	Angular Velocity	Z	Po	0.65	-11.0(2.8)	-10.0(2.3)
Canter	PARC per strid	Acceleration	H	FC left	0.5	-2.6(0.17)	-2.6(0.14)
Canter	PARC per strid	Acceleration	H	FC right	1	-2.6(0.22)	-2.6(0.15)
Canter	PARC per strid	Acceleration	H	HC left	0.84	-2.5(0.12)	-2.6(0.12)
Canter	PARC per strid	Acceleration	H	HC right	0.57	-2.1(0.091)	-2.1(0.11)
Canter	PARC per strid	Acceleration	H	FH	0.69	-2.3(0.22)	-2.4(0.2)
Canter	PARC per strid	Acceleration	H	Sa	0.84	-2.7(0.46)	-2.8(0.24)
Canter	PARC per strid	Acceleration	H	St	1	-3.0(0.39)	-3.0(0.31)
Canter	PARC per strid	Acceleration	H	Po	0.43	-2.5(0.24)	-2.4(0.24)
Canter	PARC per strid	Acceleration	X	FC left	0.25	-3.0(0.35)	-3.0(0.34)
Canter	PARC per strid	Acceleration	X	FC right	0.3	-3.0(0.29)	-2.9(0.27)
Canter	PARC per strid	Acceleration	X	HC left	1	-2.7(0.21)	-2.7(0.17)
Canter	PARC per strid	Acceleration	X	HC right	0.3	-2.7(0.072)	-2.7(0.11)
Canter	PARC per strid	Acceleration	X	FH	0.47	-2.4(0.2)	-2.3(0.25)
Canter	PARC per strid	Acceleration	X	Sa	0.56	-3.9(0.62)	-3.9(0.62)
Canter	PARC per strid	Acceleration	X	St	0.13	-4.4(0.65)	-4.6(0.72)
Canter	PARC per strid	Acceleration	X	Po	0.91	-3.0(0.32)	-3.0(0.21)
Canter	PARC per strid	Acceleration	Y	FC left	1	-4.0(0.34)	-4.0(0.32)
Canter	PARC per strid	Acceleration	Y	FC right	0.58	-3.8(0.36)	-3.9(0.28)
Canter	PARC per strid	Acceleration	Y	HC left	0.46	-3.4(0.35)	-3.4(0.33)
Canter	PARC per strid	Acceleration	Y	HC right	1	-3.4(0.29)	-3.4(0.18)
Canter	PARC per strid	Acceleration	Y	FH	0.078	-3.8(0.39)	-3.4(0.15)
Canter	PARC per strid	Acceleration	Y	Sa	0.44	-4.9(1.2)	-4.8(0.99)
Canter	PARC per strid	Acceleration	Y	St	0.91	-4.3(0.71)	-4.3(0.75)
Canter	PARC per strid	Acceleration	Y	Po	0.82	-3.1(0.22)	-3.1(0.4)
Canter	PARC per strid	Acceleration	Z	FC left	0.43	-3.4(0.4)	-3.3(0.48)
Canter	PARC per strid	Acceleration	Z	FC right	0.69	-3.4(0.55)	-3.7(0.61)
Canter	PARC per strid	Acceleration	Z	HC left	0.55	-3.7(0.43)	-3.6(0.46)
Canter	PARC per strid	Acceleration	Z	HC right	0.91	-3.8(0.58)	-3.9(0.5)
Canter	PARC per strid	Acceleration	Z	FH	0.94	-3.8(0.94)	-3.9(1.1)
Canter	PARC per strid	Acceleration	Z	Sa	0.44	-3.5(0.38)	-3.4(0.23)
Canter	PARC per strid	Acceleration	Z	St	1	-3.4(0.32)	-3.4(0.3)
Canter	PARC per strid	Acceleration	Z	Po	0.82	-2.6(0.36)	-2.6(0.39)
Canter	PARC per strid	Acceleration	V	FC left	0.36	-3.3(0.44)	-3.5(0.57)
Canter	PARC per strid	Acceleration	V	FC right	0.47	-2.9(0.34)	-3.1(0.36)
Canter	PARC per strid	Acceleration	V	HC left	1	-2.7(0.24)	-2.7(0.29)
Canter	PARC per strid	Acceleration	V	HC right	0.16	-3.2(0.17)	-3.1(0.17)
Canter	PARC per strid	Acceleration	V	FH	0.58	-2.5(0.24)	-2.4(0.29)
Canter	PARC per strid	Acceleration	V	Sa	0.44	-3.5(0.28)	-3.3(0.24)
Canter	PARC per strid	Acceleration	V	St	0.73	-3.4(0.29)	-3.4(0.33)
Canter	PARC per strid	Acceleration	V	Po	0.039	-3.2(0.56)	-3.0(0.5)
Canter	PARC per strid	Angle	Swing	FC left	0.82	-2.4(0.037)	-2.4(0.029)
Canter	PARC per strid	Angle	Swing	FC right	0.81	-2.4(0.051)	-2.4(0.051)
Canter	PARC per strid	Angle	Swing	HC left	0.55	-2.4(0.032)	-2.4(0.043)
Canter	PARC per strid	Angle	Swing	HC right	0.0039	-2.4(0.024)	-2.4(0.022)

Canter	PARC per strid	Angle	Twist	FC left	0.91	-2.2(0.17)	-2.3(0.18)
Canter	PARC per strid	Angle	Twist	FC right	0.81	-2.1(0.17)	-2.1(0.24)
Canter	PARC per strid	Angle	Twist	HC left	0.25	-2.2(0.25)	-2.3(0.23)
Canter	PARC per strid	Angle	Twist	HC right	0.43	-2.6(0.19)	-2.5(0.16)
Canter	PARC per strid	Angle	X/V	FC left	0.16	-2.4(0.026)	-2.4(0.035)
Canter	PARC per strid	Angle	X/V	FC right	0.58	-2.4(0.05)	-2.4(0.034)
Canter	PARC per strid	Angle	X/V	HC left	0.38	-2.4(0.018)	-2.4(0.041)
Canter	PARC per strid	Angle	X/V	HC right	0.074	-2.4(0.035)	-2.4(0.038)
Canter	PARC per strid	Angle	X/V	FH	0.81	-2.4(0.039)	-2.4(0.028)
Canter	PARC per strid	Angle	X/V	Sa	0.44	-2.3(0.023)	-2.3(0.018)
Canter	PARC per strid	Angle	X/V	St	0.5	-2.4(0.045)	-2.4(0.041)
Canter	PARC per strid	Angle	X/V	Po	0.2	-2.3(0.063)	-2.4(0.077)
Canter	PARC per strid	Angle	Y/V	FC left	0.43	-2.5(0.061)	-2.5(0.089)
Canter	PARC per strid	Angle	Y/V	FC right	0.81	-1.9(0.14)	-1.9(0.055)
Canter	PARC per strid	Angle	Y/V	HC left	0.74	-2.1(0.046)	-2.1(0.054)
Canter	PARC per strid	Angle	Y/V	HC right	0.16	-2.4(0.085)	-2.3(0.11)
Canter	PARC per strid	Angle	Y/V	FH	0.031	-2.4(0.0098)	-2.4(0.02)
Canter	PARC per strid	Angle	Y/V	Sa	0.84	-2.4(0.029)	-2.4(0.023)
Canter	PARC per strid	Angle	Y/V	St	0.43	-2.4(0.016)	-2.4(0.019)
Canter	PARC per strid	Angle	Y/V	Po	0.5	-2.4(0.016)	-2.4(0.024)
Canter	PARC per strid	Angle	Z/V	FC left	0.82	-2.4(0.037)	-2.4(0.029)
Canter	PARC per strid	Angle	Z/V	FC right	0.81	-2.4(0.051)	-2.4(0.051)
Canter	PARC per strid	Angle	Z/V	HC left	0.55	-2.4(0.032)	-2.4(0.043)
Canter	PARC per strid	Angle	Z/V	HC right	0.0039	-2.4(0.024)	-2.4(0.022)
Canter	PARC per strid	Angle	Z/V	FH	0.81	-2.4(0.071)	-2.4(0.057)
Canter	PARC per strid	Angle	Z/V	Sa	0.44	-2.1(0.33)	-2.1(0.24)
Canter	PARC per strid	Angle	Z/V	St	0.3	-2.4(0.022)	-2.4(0.03)
Canter	PARC per strid	Angle	Z/V	Po	0.65	-2.2(0.21)	-2.2(0.19)
Canter	PARC per strid	Angular Velocity	X	FC left	0.82	-3.1(0.66)	-3.1(0.61)
Canter	PARC per strid	Angular Velocity	X	FC right	0.94	-3.1(0.5)	-3.1(0.53)
Canter	PARC per strid	Angular Velocity	X	HC left	1	-3.6(0.56)	-3.6(0.62)
Canter	PARC per strid	Angular Velocity	X	HC right	0.65	-3.5(0.31)	-3.5(0.32)
Canter	PARC per strid	Angular Velocity	X	FH	0.38	-3.5(0.34)	-3.3(0.12)
Canter	PARC per strid	Angular Velocity	X	Sa	0.44	-4.5(0.92)	-4.3(0.75)
Canter	PARC per strid	Angular Velocity	X	St	0.055	-3.9(0.51)	-4.1(0.58)
Canter	PARC per strid	Angular Velocity	X	Po	0.91	-3.2(0.3)	-3.2(0.4)
Canter	PARC per strid	Angular Velocity	Y	FC left	0.57	-3.5(0.74)	-3.2(0.37)
Canter	PARC per strid	Angular Velocity	Y	FC right	0.3	-3.4(0.53)	-3.6(0.76)
Canter	PARC per strid	Angular Velocity	Y	HC left	0.46	-3.5(0.31)	-3.6(0.29)
Canter	PARC per strid	Angular Velocity	Y	HC right	0.73	-3.4(0.32)	-3.4(0.23)
Canter	PARC per strid	Angular Velocity	Y	FH	0.3	-3.3(0.36)	-3.1(0.19)
Canter	PARC per strid	Angular Velocity	Y	Sa	0.031	-3.3(0.64)	-3.6(0.77)
Canter	PARC per strid	Angular Velocity	Y	St	0.039	-3.2(0.22)	-3.3(0.23)
Canter	PARC per strid	Angular Velocity	Y	Po	0.73	-3.2(0.21)	-3.2(0.34)
Canter	PARC per strid	Angular Velocity	Z	FC left	0.2	-3.1(0.22)	-3.0(0.24)
Canter	PARC per strid	Angular Velocity	Z	FC right	0.3	-3.1(0.38)	-3.3(0.43)
Canter	PARC per strid	Angular Velocity	Z	HC left	0.15	-2.9(0.071)	-2.9(0.073)
Canter	PARC per strid	Angular Velocity	Z	HC right	0.16	-2.8(0.08)	-2.9(0.087)
Canter	PARC per strid	Angular Velocity	Z	FH	0.69	-3.0(0.25)	-3.0(0.31)
Canter	PARC per strid	Angular Velocity	Z	Sa	1	-4.2(0.76)	-4.2(0.52)

Canter	SPARC per strid	Angular Velocity	Z	St	1	-4.0(0.58)	-4.0(0.5)
Canter	SPARC per strid	Angular Velocity	Z	Po	0.098	-3.8(0.55)	-3.5(0.35)
Canter	SPARC per strid	Angular Velocity	Norm	FC left	0.43	-2.2(0.13)	-2.2(0.13)
Canter	SPARC per strid	Angular Velocity	Norm	FC right	0.38	-2.3(0.17)	-2.3(0.14)
Canter	SPARC per strid	Angular Velocity	Norm	HC left	0.078	-2.1(0.076)	-2.2(0.085)
Canter	SPARC per strid	Angular Velocity	Norm	HC right	1	-2.2(0.056)	-2.2(0.075)
Canter	SPARC per strid	Angular Velocity	Norm	FH	0.016	-2.3(0.071)	-2.2(0.054)
Canter	SPARC per strid	Angular Velocity	Norm	Sa	1	-2.8(0.23)	-2.8(0.17)
Canter	SPARC per strid	Angular Velocity	Norm	St	0.2	-2.5(0.12)	-2.5(0.086)
Canter	SPARC per strid	Angular Velocity	Norm	Po	0.43	-2.3(0.1)	-2.3(0.07)
Canter	SPARC	Angular Velocity	Norm	FC left	0.43	-3.7(0.26)	-3.8(0.24)
Canter	SPARC	Angular Velocity	Norm	FC right	0.58	-3.8(0.27)	-3.9(0.18)
Canter	SPARC	Angular Velocity	Norm	HC left	0.46	-3.7(0.25)	-3.6(0.24)
Canter	SPARC	Angular Velocity	Norm	HC right	0.5	-3.6(0.24)	-3.7(0.2)
Canter	SPARC	Angular Velocity	Norm	FH	0.38	-3.7(0.44)	-3.5(0.09)
Canter	SPARC	Angular Velocity	Norm	Sa	1	-4.1(0.31)	-4.0(0.22)
Canter	SPARC	Angular Velocity	Norm	St	0.36	-3.7(0.21)	-3.8(0.4)
Canter	SPARC	Angular Velocity	Norm	Po	0.3	-3.7(0.34)	-3.5(0.18)
Trot	CC	Acceleration	H	FC left	0.02	0.62(0.16)	0.37(0.25)
Trot	CC	Acceleration	H	FC right	0.016	0.66(0.15)	0.41(0.27)
Trot	CC	Acceleration	H	HC left	0.039	0.54(0.18)	0.22(0.4)
Trot	CC	Acceleration	H	HC right	0.0039	0.67(0.16)	0.45(0.32)
Trot	CC	Acceleration	H	FH	0.22	0.84(0.1)	0.81(0.086)
Trot	CC	Acceleration	H	Sa	0.031	0.53(0.14)	0.33(0.28)
Trot	CC	Acceleration	H	St	0.0078	0.6(0.13)	0.5(0.15)
Trot	CC	Acceleration	H	Po	0.039	0.61(0.14)	0.51(0.19)
Trot	CC	Acceleration	X	FC left	0.02	0.6(0.19)	0.33(0.3)
Trot	CC	Acceleration	X	FC right	0.016	0.6(0.2)	0.32(0.34)
Trot	CC	Acceleration	X	HC left	0.016	0.62(0.15)	0.34(0.33)
Trot	CC	Acceleration	X	HC right	0.0039	0.59(0.16)	0.27(0.27)
Trot	CC	Acceleration	X	FH	0.047	0.93(0.025)	0.91(0.032)
Trot	CC	Acceleration	X	Sa	0.031	0.76(0.087)	0.53(0.28)
Trot	CC	Acceleration	X	St	0.0078	0.83(0.056)	0.77(0.058)
Trot	CC	Acceleration	X	Po	0.0039	0.84(0.081)	0.76(0.13)
Trot	CC	Acceleration	Y	FC left	0.039	0.62(0.2)	0.32(0.36)
Trot	CC	Acceleration	Y	FC right	0.031	0.64(0.19)	0.28(0.45)
Trot	CC	Acceleration	Y	HC left	0.055	0.65(0.18)	0.34(0.4)
Trot	CC	Acceleration	Y	HC right	0.0039	0.62(0.18)	0.29(0.35)
Trot	CC	Acceleration	Y	FH	0.016	0.55(0.13)	0.22(0.37)
Trot	CC	Acceleration	Y	Sa	0.063	0.58(0.25)	0.35(0.38)
Trot	CC	Acceleration	Y	St	0.0078	0.57(0.21)	0.28(0.37)
Trot	CC	Acceleration	Y	Po	0.0039	0.63(0.13)	0.35(0.39)
Trot	CC	Acceleration	Z	FC left	0.0078	0.42(0.14)	0.18(0.18)
Trot	CC	Acceleration	Z	FC right	0.031	0.46(0.14)	0.21(0.25)
Trot	CC	Acceleration	Z	HC left	0.11	0.56(0.12)	0.33(0.34)
Trot	CC	Acceleration	Z	HC right	0.0039	0.56(0.13)	0.24(0.29)
Trot	CC	Acceleration	Z	FH	0.016	0.75(0.088)	0.6(0.21)
Trot	CC	Acceleration	Z	Sa	0.031	0.96(0.033)	0.93(0.05)
Trot	CC	Acceleration	Z	St	0.055	0.94(0.041)	0.93(0.04)
Trot	CC	Acceleration	Z	Po	0.0039	0.94(0.025)	0.91(0.031)

Trot	CC	Acceleration	V	FC left	0.012	0.26(0.13)	0.039(0.21)
Trot	CC	Acceleration	V	FC right	0.047	0.22(0.16)	0.02(0.19)
Trot	CC	Acceleration	V	HC left	0.055	0.2(0.16)	0.02(0.19)
Trot	CC	Acceleration	V	HC right	0.02	0.22(0.13)	0.085(0.14)
Trot	CC	Acceleration	V	FH	0.58	0.064(0.11)	0.08(0.14)
Trot	CC	Acceleration	V	Sa	0.031	0.95(0.031)	0.93(0.049)
Trot	CC	Acceleration	V	St	0.039	0.94(0.037)	0.93(0.046)
Trot	CC	Acceleration	V	Po	0.055	0.76(0.23)	0.72(0.23)
Trot	CC	Angle	Swing	FC left	0.074	0.7(0.27)	0.27(0.66)
Trot	CC	Angle	Swing	FC right	0.11	0.78(0.28)	0.4(0.57)
Trot	CC	Angle	Swing	HC left	0.46	0.78(0.27)	0.4(0.66)
Trot	CC	Angle	Swing	HC right	0.098	0.73(0.25)	0.25(0.62)
Trot	CC	Angle	Twist	FC left	0.055	0.38(0.46)	0.0096(0.62)
Trot	CC	Angle	Twist	FC right	0.078	0.69(0.37)	-0.054(0.83)
Trot	CC	Angle	Twist	HC left	0.46	0.35(0.45)	0.12(0.59)
Trot	CC	Angle	Twist	HC right	0.039	0.66(0.4)	-0.33(0.67)
Trot	CC	Angle	X/V	FC left	0.039	0.84(0.17)	0.51(0.5)
Trot	CC	Angle	X/V	FC right	0.016	0.88(0.17)	0.37(0.76)
Trot	CC	Angle	X/V	HC left	0.11	0.78(0.28)	0.27(0.72)
Trot	CC	Angle	X/V	HC right	0.36	0.84(0.23)	0.46(0.73)
Trot	CC	Angle	X/V	FH	0.81	0.19(0.14)	0.079(0.26)
Trot	CC	Angle	X/V	Sa	0.031	0.82(0.13)	0.69(0.24)
Trot	CC	Angle	X/V	St	0.0039	0.9(0.069)	0.82(0.15)
Trot	CC	Angle	X/V	Po	0.82	0.38(0.19)	0.32(0.24)
Trot	CC	Angle	Y/V	FC left	1	0.75(0.31)	0.36(0.74)
Trot	CC	Angle	Y/V	FC right	0.47	0.82(0.31)	0.29(0.85)
Trot	CC	Angle	Y/V	HC left	0.95	0.78(0.32)	0.32(0.8)
Trot	CC	Angle	Y/V	HC right	1	0.75(0.31)	0.35(0.75)
Trot	CC	Angle	Y/V	FH	0.3	0.42(0.23)	0.24(0.36)
Trot	CC	Angle	Y/V	Sa	1	0.61(0.29)	0.49(0.48)
Trot	CC	Angle	Y/V	St	0.16	0.59(0.27)	0.22(0.59)
Trot	CC	Angle	Y/V	Po	0.73	0.39(0.19)	0.24(0.33)
Trot	CC	Angle	Z/V	FC left	0.074	0.7(0.27)	0.27(0.66)
Trot	CC	Angle	Z/V	FC right	0.11	0.78(0.28)	0.4(0.57)
Trot	CC	Angle	Z/V	HC left	0.46	0.78(0.27)	0.4(0.66)
Trot	CC	Angle	Z/V	HC right	0.098	0.73(0.25)	0.25(0.62)
Trot	CC	Angle	Z/V	FH	1	0.39(0.14)	0.35(0.19)
Trot	CC	Angle	Z/V	Sa	0.031	0.7(0.19)	0.55(0.26)
Trot	CC	Angle	Z/V	St	0.0039	0.72(0.26)	0.66(0.28)
Trot	CC	Angle	Z/V	Po	0.3	0.37(0.15)	0.28(0.2)
Trot	CC	Angular Velocity	X	FC left	0.0039	0.7(0.12)	0.43(0.21)
Trot	CC	Angular Velocity	X	FC right	0.016	0.73(0.097)	0.48(0.27)
Trot	CC	Angular Velocity	X	HC left	0.039	0.74(0.12)	0.49(0.38)
Trot	CC	Angular Velocity	X	HC right	0.0039	0.71(0.11)	0.42(0.35)
Trot	CC	Angular Velocity	X	FH	0.016	0.5(0.14)	0.11(0.34)
Trot	CC	Angular Velocity	X	Sa	0.16	0.64(0.25)	0.59(0.21)
Trot	CC	Angular Velocity	X	St	0.039	0.67(0.22)	0.27(0.57)
Trot	CC	Angular Velocity	X	Po	0.0078	0.51(0.17)	0.22(0.31)
Trot	CC	Angular Velocity	Y	FC left	0.02	0.67(0.23)	0.26(0.44)
Trot	CC	Angular Velocity	Y	FC right	0.047	0.71(0.24)	0.32(0.47)

Trot	CC	Angular Velocity	Y	HC left	0.039	0.63(0.17)	0.35(0.43)
Trot	CC	Angular Velocity	Y	HC right	0.012	0.6(0.16)	0.23(0.37)
Trot	CC	Angular Velocity	Y	FH	0.031	0.64(0.13)	0.48(0.29)
Trot	CC	Angular Velocity	Y	Sa	0.031	0.84(0.085)	0.76(0.12)
Trot	CC	Angular Velocity	Y	St	0.0078	0.89(0.075)	0.83(0.13)
Trot	CC	Angular Velocity	Y	Po	0.02	0.56(0.18)	0.4(0.32)
Trot	CC	Angular Velocity	Z	FC left	0.43	0.76(0.26)	0.42(0.6)
Trot	CC	Angular Velocity	Z	FC right	0.16	0.82(0.25)	0.37(0.71)
Trot	CC	Angular Velocity	Z	HC left	0.078	0.78(0.27)	0.33(0.65)
Trot	CC	Angular Velocity	Z	HC right	0.43	0.75(0.26)	0.43(0.58)
Trot	CC	Angular Velocity	Z	FH	0.11	0.54(0.24)	0.3(0.34)
Trot	CC	Angular Velocity	Z	Sa	0.31	0.58(0.25)	0.5(0.19)
Trot	CC	Angular Velocity	Z	St	0.098	0.7(0.28)	0.22(0.7)
Trot	CC	Angular Velocity	Z	Po	0.0078	0.51(0.16)	0.13(0.37)
Trot	Duration				0.65	0.84(0.043)	0.83(0.049)
Trot	LDLJ-A			FC left	0.73	-14.0(0.58)	-14.0(0.31)
Trot	LDLJ-A			FC right	0.47	-14.0(0.6)	-14.0(0.29)
Trot	LDLJ-A			HC left	0.46	-13.0(0.62)	-13.0(0.45)
Trot	LDLJ-A			HC right	0.43	-13.0(0.56)	-13.0(0.39)
Trot	LDLJ-A			FH	0.69	-10.0(0.43)	-10.0(0.32)
Trot	LDLJ-A			Sa	1	-12.0(0.44)	-12.0(0.41)
Trot	LDLJ-A			St	0.65	-11.0(0.55)	-11.0(0.32)
Trot	LDLJ-A			Po	0.65	-11.0(0.4)	-11.0(0.3)
Trot	LDLJ-A			FC left	1	-10.0(0.24)	-10.0(0.21)
Trot	LDLJ-A			FC right	0.22	-10.0(0.2)	-10.0(0.21)
Trot	LDLJ-A			HC left	0.84	-9.7(0.24)	-9.7(0.24)
Trot	LDLJ-A			HC right	0.82	-9.9(0.21)	-9.9(0.23)
Trot	LDLJ-A			FH	0.3	-7.1(0.32)	-7.2(0.23)
Trot	LDLJ-A			Sa	0.063	-8.3(0.21)	-8.2(0.14)
Trot	LDLJ-A			St	1	-8.1(0.26)	-8.1(0.25)
Trot	LDLJ-A			Po	0.36	-7.4(0.22)	-7.5(0.27)
Trot	RMSD	Acceleration	H	FC left	0.012	13.0(2.4)	18.0(4.2)
Trot	RMSD	Acceleration	H	FC right	0.016	13.0(2.7)	17.0(4.0)
Trot	RMSD	Acceleration	H	HC left	0.016	10.0(2.0)	14.0(4.6)
Trot	RMSD	Acceleration	H	HC right	0.0039	10.0(2.2)	14.0(4.3)
Trot	RMSD	Acceleration	H	FH	0.016	2.7(0.95)	3.2(1.0)
Trot	RMSD	Acceleration	H	Sa	0.031	2.8(0.15)	3.4(0.31)
Trot	RMSD	Acceleration	H	St	0.0078	2.7(0.45)	3.1(0.51)
Trot	RMSD	Acceleration	H	Po	0.0078	4.5(1.1)	5.1(0.95)
Trot	RMSD	Acceleration	X	FC left	0.02	13.0(3.3)	17.0(5.2)
Trot	RMSD	Acceleration	X	FC right	0.016	12.0(3.0)	17.0(5.7)
Trot	RMSD	Acceleration	X	HC left	0.016	11.0(2.8)	15.0(5.3)
Trot	RMSD	Acceleration	X	HC right	0.0039	11.0(2.8)	16.0(3.9)
Trot	RMSD	Acceleration	X	FH	0.016	3.1(0.55)	3.6(0.65)
Trot	RMSD	Acceleration	X	Sa	0.031	2.2(0.31)	3.0(0.81)
Trot	RMSD	Acceleration	X	St	0.0078	2.7(0.51)	3.2(0.55)
Trot	RMSD	Acceleration	X	Po	0.0039	3.3(0.54)	4.2(0.79)
Trot	RMSD	Acceleration	Y	FC left	0.027	21.0(5.1)	30.0(9.0)
Trot	RMSD	Acceleration	Y	FC right	0.016	20.0(4.7)	29.0(11.0)
Trot	RMSD	Acceleration	Y	HC left	0.039	15.0(4.1)	21.0(8.1)

Trot	RMSD	Acceleration	Y	HC right	0.0039	16.0(3.8)	23.0(7.3)
Trot	RMSD	Acceleration	Y	FH	0.016	2.2(0.57)	2.8(0.98)
Trot	RMSD	Acceleration	Y	Sa	0.063	3.2(1.0)	4.5(1.7)
Trot	RMSD	Acceleration	Y	St	0.0039	2.3(0.48)	3.1(1.0)
Trot	RMSD	Acceleration	Y	Po	0.0039	2.3(0.39)	3.0(0.82)
Trot	RMSD	Acceleration	Z	FC left	0.0078	9.8(2.0)	12.0(2.4)
Trot	RMSD	Acceleration	Z	FC right	0.016	8.7(1.4)	11.0(1.6)
Trot	RMSD	Acceleration	Z	HC left	0.039	8.3(1.4)	10.0(3.2)
Trot	RMSD	Acceleration	Z	HC right	0.0039	9.0(1.6)	12.0(2.5)
Trot	RMSD	Acceleration	Z	FH	0.016	2.0(0.45)	2.7(0.79)
Trot	RMSD	Acceleration	Z	Sa	0.031	2.0(0.5)	2.7(0.59)
Trot	RMSD	Acceleration	Z	St	0.012	2.1(0.44)	2.4(0.48)
Trot	RMSD	Acceleration	Z	Po	0.0039	2.1(0.44)	2.7(0.37)
Trot	RMSD	Acceleration	V	FC left	0.0078	27.0(2.5)	31.0(4.7)
Trot	RMSD	Acceleration	V	FC right	0.031	26.0(3.1)	29.0(3.5)
Trot	RMSD	Acceleration	V	HC left	0.039	22.0(1.4)	25.0(3.4)
Trot	RMSD	Acceleration	V	HC right	0.0039	23.0(2.2)	26.0(2.5)
Trot	RMSD	Acceleration	V	FH	0.81	11.0(1.9)	11.0(2.3)
Trot	RMSD	Acceleration	V	Sa	0.031	2.2(0.42)	2.8(0.54)
Trot	RMSD	Acceleration	V	St	0.02	2.2(0.33)	2.5(0.42)
Trot	RMSD	Acceleration	V	Po	0.02	4.4(2.1)	5.0(2.0)
Trot	RMSD	Angle	Swing	FC left	0.012	4.9(2.0)	6.8(2.4)
Trot	RMSD	Angle	Swing	FC right	0.078	5.2(2.4)	6.8(2.8)
Trot	RMSD	Angle	Swing	HC left	0.078	4.5(1.2)	7.2(4.6)
Trot	RMSD	Angle	Swing	HC right	0.02	4.5(0.91)	7.0(2.9)
Trot	RMSD	Angle	Twist	FC left	0.0078	69.0(16.0)	150.0(76.0)
Trot	RMSD	Angle	Twist	FC right	0.016	88.0(11.0)	120.0(41.0)
Trot	RMSD	Angle	Twist	HC left	0.039	51.0(19.0)	110.0(67.0)
Trot	RMSD	Angle	Twist	HC right	0.055	83.0(12.0)	110.0(51.0)
Trot	RMSD	Angle	X/V	FC left	0.0039	5.7(2.7)	12.0(7.6)
Trot	RMSD	Angle	X/V	FC right	0.016	5.6(2.8)	17.0(16.0)
Trot	RMSD	Angle	X/V	HC left	0.0078	5.0(2.8)	9.4(5.9)
Trot	RMSD	Angle	X/V	HC right	0.074	4.2(2.2)	8.2(7.7)
Trot	RMSD	Angle	X/V	FH	0.11	4.5(1.4)	5.6(2.3)
Trot	RMSD	Angle	X/V	Sa	0.16	2.1(0.61)	3.1(1.5)
Trot	RMSD	Angle	X/V	St	0.0039	2.3(0.52)	3.7(1.6)
Trot	RMSD	Angle	X/V	Po	0.055	5.0(0.79)	7.3(4.3)
Trot	RMSD	Angle	Y/V	FC left	0.074	10.0(7.7)	22.0(19.0)
Trot	RMSD	Angle	Y/V	FC right	0.016	8.4(7.7)	25.0(21.0)
Trot	RMSD	Angle	Y/V	HC left	0.11	6.8(5.5)	16.0(15.0)
Trot	RMSD	Angle	Y/V	HC right	0.2	7.3(5.1)	15.0(14.0)
Trot	RMSD	Angle	Y/V	FH	0.078	3.6(0.8)	4.0(1.1)
Trot	RMSD	Angle	Y/V	Sa	0.031	4.9(1.3)	6.0(1.8)
Trot	RMSD	Angle	Y/V	St	0.055	3.7(1.1)	5.4(2.0)
Trot	RMSD	Angle	Y/V	Po	0.02	3.4(0.77)	4.7(2.6)
Trot	RMSD	Angle	Z/V	FC left	0.012	4.9(2.0)	6.8(2.4)
Trot	RMSD	Angle	Z/V	FC right	0.078	5.2(2.4)	6.8(2.8)
Trot	RMSD	Angle	Z/V	HC left	0.078	4.5(1.2)	7.2(4.6)
Trot	RMSD	Angle	Z/V	HC right	0.02	4.5(0.91)	7.0(2.9)
Trot	RMSD	Angle	Z/V	FH	0.031	5.5(1.2)	8.2(5.0)

Trot	RMSD	Angle	Z/V	Sa	0.031	2.5(0.72)	3.7(1.5)
Trot	RMSD	Angle	Z/V	St	0.0039	2.6(0.48)	4.4(1.7)
Trot	RMSD	Angle	Z/V	Po	0.0078	4.9(0.87)	7.7(3.6)
Trot	RMSD	Angular Velocity	X	FC left	0.0039	1.8(0.48)	2.6(0.59)
Trot	RMSD	Angular Velocity	X	FC right	0.016	1.7(0.44)	2.4(0.87)
Trot	RMSD	Angular Velocity	X	HC left	0.0078	1.3(0.36)	1.9(0.85)
Trot	RMSD	Angular Velocity	X	HC right	0.0039	1.3(0.31)	1.9(0.67)
Trot	RMSD	Angular Velocity	X	FH	0.016	0.47(0.14)	0.63(0.27)
Trot	RMSD	Angular Velocity	X	Sa	0.16	0.86(0.45)	1.0(0.42)
Trot	RMSD	Angular Velocity	X	St	0.0039	0.36(0.15)	0.56(0.29)
Trot	RMSD	Angular Velocity	X	Po	0.0039	0.36(0.078)	0.45(0.11)
Trot	RMSD	Angular Velocity	Y	FC left	0.0078	0.83(0.37)	1.3(0.41)
Trot	RMSD	Angular Velocity	Y	FC right	0.031	0.78(0.29)	1.2(0.41)
Trot	RMSD	Angular Velocity	Y	HC left	0.023	0.67(0.2)	0.93(0.48)
Trot	RMSD	Angular Velocity	Y	HC right	0.0039	0.71(0.16)	1.0(0.27)
Trot	RMSD	Angular Velocity	Y	FH	0.031	0.44(0.11)	0.53(0.23)
Trot	RMSD	Angular Velocity	Y	Sa	0.031	0.25(0.038)	0.33(0.037)
Trot	RMSD	Angular Velocity	Y	St	0.0078	0.29(0.065)	0.39(0.1)
Trot	RMSD	Angular Velocity	Y	Po	0.055	0.42(0.1)	0.48(0.19)
Trot	RMSD	Angular Velocity	Z	FC left	0.074	1.8(0.99)	3.4(2.4)
Trot	RMSD	Angular Velocity	Z	FC right	0.031	1.6(1.0)	3.6(2.8)
Trot	RMSD	Angular Velocity	Z	HC left	0.055	1.3(0.72)	2.6(1.9)
Trot	RMSD	Angular Velocity	Z	HC right	0.02	1.4(0.67)	2.4(1.7)
Trot	RMSD	Angular Velocity	Z	FH	0.016	0.29(0.07)	0.36(0.1)
Trot	RMSD	Angular Velocity	Z	Sa	0.063	0.22(0.09)	0.27(0.11)
Trot	RMSD	Angular Velocity	Z	St	0.02	0.32(0.13)	0.59(0.38)
Trot	RMSD	Angular Velocity	Z	Po	0.0039	0.38(0.099)	0.5(0.18)
Trot	SPARC	Acceleration	H	FC left	1	-5.0(0.51)	-5.0(0.45)
Trot	SPARC	Acceleration	H	FC right	0.16	-4.9(0.72)	-5.1(0.55)
Trot	SPARC	Acceleration	H	HC left	0.55	-4.1(0.36)	-4.3(0.38)
Trot	SPARC	Acceleration	H	HC right	0.73	-5.6(0.71)	-5.5(0.66)
Trot	SPARC	Acceleration	H	FH	0.81	-4.9(0.72)	-4.8(0.41)
Trot	SPARC	Acceleration	H	Sa	0.44	-4.1(0.26)	-4.3(0.3)
Trot	SPARC	Acceleration	H	St	0.91	-4.4(0.52)	-4.4(0.41)
Trot	SPARC	Acceleration	H	Po	0.82	-4.7(0.69)	-4.8(0.22)
Trot	SPARC	Acceleration	X	FC left	0.2	-5.5(0.72)	-5.8(0.32)
Trot	SPARC	Acceleration	X	FC right	0.16	-5.6(0.83)	-5.8(0.52)
Trot	SPARC	Acceleration	X	HC left	0.31	-5.3(0.65)	-5.6(0.61)
Trot	SPARC	Acceleration	X	HC right	1	-5.4(0.74)	-5.4(0.58)
Trot	SPARC	Acceleration	X	FH	0.94	-5.3(0.62)	-5.3(0.35)
Trot	SPARC	Acceleration	X	Sa	1	-9.0(1.0)	-9.0(0.82)
Trot	SPARC	Acceleration	X	St	0.43	-9.4(2.2)	-9.7(1.7)
Trot	SPARC	Acceleration	X	Po	0.91	-8.5(2.1)	-8.5(1.1)
Trot	SPARC	Acceleration	Y	FC left	0.3	-11.0(2.2)	-12.0(1.7)
Trot	SPARC	Acceleration	Y	FC right	0.38	-11.0(2.0)	-11.0(1.3)
Trot	SPARC	Acceleration	Y	HC left	0.15	-11.0(1.8)	-12.0(1.2)
Trot	SPARC	Acceleration	Y	HC right	1	-12.0(2.0)	-12.0(1.4)
Trot	SPARC	Acceleration	Y	FH	0.94	-12.0(2.1)	-13.0(2.1)
Trot	SPARC	Acceleration	Y	Sa	1	-11.0(1.6)	-11.0(1.3)
Trot	SPARC	Acceleration	Y	St	0.3	-11.0(2.2)	-10.0(2.3)

Trot	SPARC	Acceleration	Y	Po	0.25	-10.0(2.4)	-11.0(2.4)
Trot	SPARC	Acceleration	Z	FC left	0.91	-12.0(3.7)	-12.0(2.0)
Trot	SPARC	Acceleration	Z	FC right	0.69	-12.0(3.9)	-12.0(3.0)
Trot	SPARC	Acceleration	Z	HC left	0.64	-13.0(2.3)	-13.0(1.6)
Trot	SPARC	Acceleration	Z	HC right	1	-14.0(2.1)	-14.0(2.1)
Trot	SPARC	Acceleration	Z	FH	0.81	-8.8(2.1)	-8.7(1.8)
Trot	SPARC	Acceleration	Z	Sa	1	-5.3(0.3)	-5.3(0.42)
Trot	SPARC	Acceleration	Z	St	1	-4.8(0.24)	-4.8(0.4)
Trot	SPARC	Acceleration	Z	Po	0.055	-4.9(0.44)	-5.1(0.56)
Trot	SPARC	Acceleration	V	FC left	0.039	-7.5(1.8)	-8.5(1.6)
Trot	SPARC	Acceleration	V	FC right	0.11	-7.8(2.0)	-8.9(1.7)
Trot	SPARC	Acceleration	V	HC left	0.078	-11.0(1.2)	-11.0(1.2)
Trot	SPARC	Acceleration	V	HC right	0.91	-8.2(2.3)	-8.3(2.6)
Trot	SPARC	Acceleration	V	FH	0.81	-5.6(0.84)	-5.4(0.47)
Trot	SPARC	Acceleration	V	Sa	0.69	-5.3(0.34)	-5.4(0.44)
Trot	SPARC	Acceleration	V	St	0.57	-4.9(0.3)	-4.9(0.35)
Trot	SPARC	Acceleration	V	Po	0.91	-5.5(1.1)	-5.5(1.0)
Trot	SPARC	Angle	Swing	FC left	0.73	-2.5(0.087)	-2.5(0.092)
Trot	SPARC	Angle	Swing	FC right	0.81	-2.4(0.12)	-2.5(0.15)
Trot	SPARC	Angle	Swing	HC left	0.55	-2.5(0.089)	-2.4(0.068)
Trot	SPARC	Angle	Swing	HC right	0.25	-2.5(0.1)	-2.4(0.032)
Trot	SPARC	Angle	Twist	FC left	0.82	-2.8(0.4)	-2.8(0.35)
Trot	SPARC	Angle	Twist	FC right	0.47	-2.8(0.37)	-2.6(0.1)
Trot	SPARC	Angle	Twist	HC left	0.46	-2.5(0.17)	-2.5(0.097)
Trot	SPARC	Angle	Twist	HC right	0.13	-2.8(0.26)	-2.6(0.13)
Trot	SPARC	Angle	X/V	FC left	0.16	-2.6(0.11)	-2.7(0.13)
Trot	SPARC	Angle	X/V	FC right	1	-2.7(0.12)	-2.7(0.13)
Trot	SPARC	Angle	X/V	HC left	0.38	-2.5(0.12)	-2.5(0.053)
Trot	SPARC	Angle	X/V	HC right	0.91	-2.5(0.11)	-2.5(0.078)
Trot	SPARC	Angle	X/V	FH	0.22	-2.4(0.038)	-2.4(0.01)
Trot	SPARC	Angle	X/V	Sa	0.44	-2.4(0.034)	-2.4(0.042)
Trot	SPARC	Angle	X/V	St	1	-2.5(0.094)	-2.5(0.071)
Trot	SPARC	Angle	X/V	Po	0.16	-2.4(0.069)	-2.4(0.061)
Trot	SPARC	Angle	Y/V	FC left	0.43	-2.9(0.25)	-3.0(0.13)
Trot	SPARC	Angle	Y/V	FC right	0.3	-2.8(0.26)	-2.9(0.12)
Trot	SPARC	Angle	Y/V	HC left	0.2	-2.6(0.15)	-2.6(0.12)
Trot	SPARC	Angle	Y/V	HC right	0.5	-2.7(0.12)	-2.7(0.12)
Trot	SPARC	Angle	Y/V	FH	0.47	-2.5(0.058)	-2.4(0.049)
Trot	SPARC	Angle	Y/V	Sa	0.31	-2.4(0.14)	-2.5(0.075)
Trot	SPARC	Angle	Y/V	St	0.57	-2.4(0.057)	-2.4(0.051)
Trot	SPARC	Angle	Y/V	Po	0.36	-2.4(0.032)	-2.4(0.067)
Trot	SPARC	Angle	Z/V	FC left	0.73	-2.5(0.087)	-2.5(0.092)
Trot	SPARC	Angle	Z/V	FC right	0.81	-2.4(0.12)	-2.5(0.15)
Trot	SPARC	Angle	Z/V	HC left	0.55	-2.5(0.089)	-2.4(0.068)
Trot	SPARC	Angle	Z/V	HC right	0.25	-2.5(0.1)	-2.4(0.032)
Trot	SPARC	Angle	Z/V	FH	0.11	-2.4(0.07)	-2.4(0.053)
Trot	SPARC	Angle	Z/V	Sa	0.16	-2.9(0.5)	-3.1(0.8)
Trot	SPARC	Angle	Z/V	St	0.3	-2.4(0.02)	-2.4(0.034)
Trot	SPARC	Angle	Z/V	Po	0.65	-2.5(0.14)	-2.6(0.41)
Trot	SPARC	Angular Velocity	X	FC left	0.43	-14.0(2.5)	-15.0(2.1)

Trot	SPARC	Angular Velocity	X	FC right	0.3	-14.0(3.3)	-15.0(2.1)
Trot	SPARC	Angular Velocity	X	HC left	0.64	-13.0(2.5)	-13.0(2.3)
Trot	SPARC	Angular Velocity	X	HC right	0.65	-14.0(1.8)	-14.0(3.0)
Trot	SPARC	Angular Velocity	X	FH	0.81	-11.0(1.9)	-12.0(1.6)
Trot	SPARC	Angular Velocity	X	Sa	0.84	-10.0(1.7)	-10.0(2.1)
Trot	SPARC	Angular Velocity	X	St	0.57	-12.0(1.9)	-12.0(1.4)
Trot	SPARC	Angular Velocity	X	Po	0.57	-11.0(2.4)	-12.0(1.7)
Trot	SPARC	Angular Velocity	Y	FC left	0.65	-11.0(2.1)	-10.0(2.4)
Trot	SPARC	Angular Velocity	Y	FC right	0.69	-11.0(2.6)	-10.0(1.6)
Trot	SPARC	Angular Velocity	Y	HC left	0.38	-12.0(2.1)	-13.0(1.7)
Trot	SPARC	Angular Velocity	Y	HC right	0.82	-11.0(2.6)	-12.0(3.2)
Trot	SPARC	Angular Velocity	Y	FH	0.69	-9.7(3.6)	-9.9(2.3)
Trot	SPARC	Angular Velocity	Y	Sa	0.44	-8.3(1.8)	-8.5(1.1)
Trot	SPARC	Angular Velocity	Y	St	0.055	-7.3(1.2)	-7.8(1.1)
Trot	SPARC	Angular Velocity	Y	Po	0.36	-9.9(3.6)	-10.0(3.0)
Trot	SPARC	Angular Velocity	Z	FC left	0.5	-6.9(0.91)	-7.2(0.71)
Trot	SPARC	Angular Velocity	Z	FC right	0.38	-6.7(0.92)	-7.0(0.4)
Trot	SPARC	Angular Velocity	Z	HC left	0.64	-7.2(0.84)	-7.3(0.54)
Trot	SPARC	Angular Velocity	Z	HC right	0.25	-7.3(1.1)	-7.6(1.0)
Trot	SPARC	Angular Velocity	Z	FH	0.94	-9.9(2.0)	-10.0(1.6)
Trot	SPARC	Angular Velocity	Z	Sa	0.063	-7.9(1.0)	-8.6(1.6)
Trot	SPARC	Angular Velocity	Z	St	0.82	-7.8(1.1)	-7.9(1.1)
Trot	SPARC	Angular Velocity	Z	Po	0.25	-13.0(2.1)	-12.0(1.6)
Trot	PARC per strid	Acceleration	H	FC left	0.57	-2.8(0.29)	-2.7(0.29)
Trot	PARC per strid	Acceleration	H	FC right	0.16	-2.5(0.18)	-2.7(0.25)
Trot	PARC per strid	Acceleration	H	HC left	0.84	-2.3(0.26)	-2.4(0.42)
Trot	PARC per strid	Acceleration	H	HC right	0.91	-2.3(0.22)	-2.3(0.24)
Trot	PARC per strid	Acceleration	H	FH	0.11	-3.4(0.34)	-3.2(0.39)
Trot	PARC per strid	Acceleration	H	Sa	1	-2.7(0.099)	-2.7(0.11)
Trot	PARC per strid	Acceleration	H	St	0.82	-2.8(0.44)	-2.8(0.26)
Trot	PARC per strid	Acceleration	H	Po	0.65	-3.1(0.35)	-3.1(0.26)
Trot	PARC per strid	Acceleration	X	FC left	0.13	-2.9(0.29)	-3.0(0.27)
Trot	PARC per strid	Acceleration	X	FC right	0.94	-2.6(0.29)	-2.7(0.3)
Trot	PARC per strid	Acceleration	X	HC left	0.74	-2.6(0.37)	-2.6(0.46)
Trot	PARC per strid	Acceleration	X	HC right	1	-2.8(0.35)	-2.7(0.41)
Trot	PARC per strid	Acceleration	X	FH	1	-3.8(0.34)	-3.8(0.42)
Trot	PARC per strid	Acceleration	X	Sa	0.56	-4.6(0.32)	-4.5(0.41)
Trot	PARC per strid	Acceleration	X	St	1	-4.8(0.8)	-4.9(0.67)
Trot	PARC per strid	Acceleration	X	Po	0.73	-4.6(0.55)	-4.7(0.31)
Trot	PARC per strid	Acceleration	Y	FC left	0.5	-4.9(1.0)	-5.0(1.0)
Trot	PARC per strid	Acceleration	Y	FC right	0.078	-4.2(0.4)	-4.5(0.42)
Trot	PARC per strid	Acceleration	Y	HC left	0.46	-3.4(0.48)	-3.6(0.89)
Trot	PARC per strid	Acceleration	Y	HC right	0.91	-4.1(0.71)	-3.9(0.59)
Trot	PARC per strid	Acceleration	Y	FH	0.078	-4.7(0.43)	-4.1(0.4)
Trot	PARC per strid	Acceleration	Y	Sa	0.56	-5.0(0.66)	-5.1(0.6)
Trot	PARC per strid	Acceleration	Y	St	0.02	-4.5(0.75)	-4.0(0.64)
Trot	PARC per strid	Acceleration	Y	Po	0.82	-4.1(0.46)	-4.2(0.38)
Trot	PARC per strid	Acceleration	Z	FC left	0.65	-3.7(0.87)	-3.7(0.72)
Trot	PARC per strid	Acceleration	Z	FC right	0.11	-4.3(0.71)	-4.0(0.6)
Trot	PARC per strid	Acceleration	Z	HC left	0.25	-3.5(0.77)	-3.7(0.77)

Trot	PARC per strid	Acceleration	Z	HC right	0.039	-4.1(0.46)	-3.8(0.58)
Trot	PARC per strid	Acceleration	Z	FH	1	-4.3(0.63)	-4.3(0.41)
Trot	PARC per strid	Acceleration	Z	Sa	0.31	-3.9(0.25)	-3.8(0.27)
Trot	PARC per strid	Acceleration	Z	St	0.3	-3.6(0.25)	-3.5(0.24)
Trot	PARC per strid	Acceleration	Z	Po	1	-3.5(0.31)	-3.5(0.39)
Trot	PARC per strid	Acceleration	V	FC left	0.25	-3.1(0.43)	-3.3(0.57)
Trot	PARC per strid	Acceleration	V	FC right	0.38	-2.9(0.54)	-3.3(0.76)
Trot	PARC per strid	Acceleration	V	HC left	0.11	-3.2(0.36)	-3.0(0.35)
Trot	PARC per strid	Acceleration	V	HC right	0.82	-3.1(0.52)	-3.1(0.64)
Trot	PARC per strid	Acceleration	V	FH	0.81	-3.7(0.3)	-3.8(0.39)
Trot	PARC per strid	Acceleration	V	Sa	0.31	-3.9(0.24)	-3.8(0.28)
Trot	PARC per strid	Acceleration	V	St	0.57	-3.6(0.25)	-3.6(0.24)
Trot	PARC per strid	Acceleration	V	Po	0.43	-3.7(0.42)	-3.6(0.37)
Trot	PARC per strid	Angle	Swing	FC left	0.43	-2.5(0.092)	-2.5(0.1)
Trot	PARC per strid	Angle	Swing	FC right	0.38	-2.4(0.037)	-2.4(0.094)
Trot	PARC per strid	Angle	Swing	HC left	1	-2.3(0.059)	-2.3(0.061)
Trot	PARC per strid	Angle	Swing	HC right	0.82	-2.4(0.043)	-2.4(0.068)
Trot	PARC per strid	Angle	Twist	FC left	0.57	-2.3(0.1)	-2.3(0.11)
Trot	PARC per strid	Angle	Twist	FC right	0.81	-2.2(0.26)	-2.2(0.14)
Trot	PARC per strid	Angle	Twist	HC left	0.55	-2.4(0.057)	-2.4(0.07)
Trot	PARC per strid	Angle	Twist	HC right	0.0039	-2.1(0.14)	-2.4(0.072)
Trot	PARC per strid	Angle	X/V	FC left	0.65	-2.5(0.14)	-2.5(0.11)
Trot	PARC per strid	Angle	X/V	FC right	0.81	-2.3(0.19)	-2.3(0.21)
Trot	PARC per strid	Angle	X/V	HC left	0.95	-2.4(0.081)	-2.4(0.091)
Trot	PARC per strid	Angle	X/V	HC right	0.65	-2.4(0.085)	-2.4(0.07)
Trot	PARC per strid	Angle	X/V	FH	0.81	-2.4(0.016)	-2.4(0.016)
Trot	PARC per strid	Angle	X/V	Sa	0.84	-2.4(0.072)	-2.4(0.074)
Trot	PARC per strid	Angle	X/V	St	0.055	-2.5(0.036)	-2.5(0.051)
Trot	PARC per strid	Angle	X/V	Po	1	-2.4(0.04)	-2.4(0.039)
Trot	PARC per strid	Angle	Y/V	FC left	0.65	-2.2(0.36)	-2.2(0.32)
Trot	PARC per strid	Angle	Y/V	FC right	1	-2.4(0.39)	-2.4(0.31)
Trot	PARC per strid	Angle	Y/V	HC left	0.84	-2.4(0.093)	-2.4(0.07)
Trot	PARC per strid	Angle	Y/V	HC right	0.2	-2.1(0.13)	-2.1(0.084)
Trot	PARC per strid	Angle	Y/V	FH	0.3	-2.4(0.024)	-2.4(0.02)
Trot	PARC per strid	Angle	Y/V	Sa	0.31	-2.5(0.12)	-2.4(0.085)
Trot	PARC per strid	Angle	Y/V	St	0.57	-2.5(0.058)	-2.5(0.065)
Trot	PARC per strid	Angle	Y/V	Po	0.82	-2.4(0.045)	-2.4(0.027)
Trot	PARC per strid	Angle	Z/V	FC left	0.43	-2.5(0.092)	-2.5(0.1)
Trot	PARC per strid	Angle	Z/V	FC right	0.38	-2.4(0.037)	-2.4(0.094)
Trot	PARC per strid	Angle	Z/V	HC left	1	-2.3(0.059)	-2.3(0.061)
Trot	PARC per strid	Angle	Z/V	HC right	0.82	-2.4(0.043)	-2.4(0.068)
Trot	PARC per strid	Angle	Z/V	FH	0.38	-2.4(0.036)	-2.4(0.03)
Trot	PARC per strid	Angle	Z/V	Sa	0.69	-2.4(0.37)	-2.3(0.2)
Trot	PARC per strid	Angle	Z/V	St	0.2	-2.4(0.052)	-2.4(0.032)
Trot	PARC per strid	Angle	Z/V	Po	0.3	-2.4(0.11)	-2.3(0.15)
Trot	PARC per strid	Angular Velocity	X	FC left	0.82	-4.5(1.5)	-4.3(1.1)
Trot	PARC per strid	Angular Velocity	X	FC right	0.47	-5.3(1.9)	-5.9(1.4)
Trot	PARC per strid	Angular Velocity	X	HC left	0.15	-4.9(0.86)	-4.5(0.93)
Trot	PARC per strid	Angular Velocity	X	HC right	0.3	-4.8(0.84)	-4.5(0.95)
Trot	PARC per strid	Angular Velocity	X	FH	0.58	-4.1(0.64)	-3.9(0.48)

Trot	PARC per strid	Angular Velocity	X	Sa	0.69	-4.9(0.55)	-4.8(0.48)
Trot	PARC per strid	Angular Velocity	X	St	0.039	-4.6(0.45)	-4.4(0.31)
Trot	PARC per strid	Angular Velocity	X	Po	0.73	-4.0(0.4)	-4.0(0.67)
Trot	PARC per strid	Angular Velocity	Y	FC left	0.36	-3.7(1.0)	-3.5(0.74)
Trot	PARC per strid	Angular Velocity	Y	FC right	0.38	-4.2(0.9)	-3.9(0.97)
Trot	PARC per strid	Angular Velocity	Y	HC left	0.55	-4.7(0.84)	-5.0(0.78)
Trot	PARC per strid	Angular Velocity	Y	HC right	1	-4.5(0.85)	-4.2(0.65)
Trot	PARC per strid	Angular Velocity	Y	FH	0.3	-4.0(0.53)	-3.8(0.44)
Trot	PARC per strid	Angular Velocity	Y	Sa	0.44	-4.1(0.45)	-4.1(0.3)
Trot	PARC per strid	Angular Velocity	Y	St	0.57	-3.8(0.32)	-3.9(0.26)
Trot	PARC per strid	Angular Velocity	Y	Po	0.055	-3.8(0.55)	-3.6(0.42)
Trot	PARC per strid	Angular Velocity	Z	FC left	0.91	-3.7(0.93)	-3.7(0.99)
Trot	PARC per strid	Angular Velocity	Z	FC right	0.58	-3.8(0.82)	-4.0(0.85)
Trot	PARC per strid	Angular Velocity	Z	HC left	0.95	-3.3(0.21)	-3.3(0.26)
Trot	PARC per strid	Angular Velocity	Z	HC right	1	-3.3(0.15)	-3.3(0.25)
Trot	PARC per strid	Angular Velocity	Z	FH	0.031	-3.9(0.46)	-3.6(0.41)
Trot	PARC per strid	Angular Velocity	Z	Sa	0.69	-3.9(0.51)	-3.8(0.61)
Trot	PARC per strid	Angular Velocity	Z	St	0.36	-3.6(0.39)	-3.7(0.45)
Trot	PARC per strid	Angular Velocity	Z	Po	0.2	-4.2(0.46)	-4.0(0.4)
Trot	PARC per strid	Angular Velocity	Norm	FC left	1	-2.4(0.46)	-2.4(0.42)
Trot	PARC per strid	Angular Velocity	Norm	FC right	0.38	-2.5(0.41)	-2.6(0.41)
Trot	PARC per strid	Angular Velocity	Norm	HC left	0.46	-2.4(0.2)	-2.5(0.2)
Trot	PARC per strid	Angular Velocity	Norm	HC right	0.73	-2.4(0.13)	-2.4(0.22)
Trot	PARC per strid	Angular Velocity	Norm	FH	0.11	-2.6(0.22)	-2.4(0.057)
Trot	PARC per strid	Angular Velocity	Norm	Sa	0.69	-2.8(0.24)	-2.8(0.3)
Trot	PARC per strid	Angular Velocity	Norm	St	0.36	-2.5(0.1)	-2.5(0.12)
Trot	PARC per strid	Angular Velocity	Norm	Po	0.5	-2.5(0.12)	-2.4(0.12)
Trot	SPARC	Angular Velocity	Norm	FC left	0.43	-4.3(0.58)	-4.4(0.4)
Trot	SPARC	Angular Velocity	Norm	FC right	0.58	-4.2(0.64)	-4.4(0.086)
Trot	SPARC	Angular Velocity	Norm	HC left	0.2	-4.0(0.31)	-4.2(0.26)
Trot	SPARC	Angular Velocity	Norm	HC right	0.91	-4.0(0.37)	-4.0(0.26)
Trot	SPARC	Angular Velocity	Norm	FH	0.58	-3.8(0.27)	-4.0(0.29)
Trot	SPARC	Angular Velocity	Norm	Sa	0.56	-4.9(0.39)	-5.1(0.52)
Trot	SPARC	Angular Velocity	Norm	St	0.82	-4.1(0.39)	-4.1(0.38)
Trot	SPARC	Angular Velocity	Norm	Po	0.43	-3.6(0.29)	-3.8(0.27)
